# Genetic Diversity, Population Structure, and Cross-Border Dispersal Patterns of Tomato Leaf Curl Palampur Virus in South and West Asia

**DOI:** 10.3390/v17050678

**Published:** 2025-05-06

**Authors:** Muhammad Naeem Sattar, Biju V. Chellappan, Sherif M. ElGanainy, Mustafa I. Almaghaslah, Sallah A. Al Hashedi, Adil A. Al-Shoaibi

**Affiliations:** 1 Central Laboratories, King Faisal University, P.O. Box 420, Al-Ahsa 31982, Saudi Arabia; sahmad@kfu.edu.sa (S.A.A.H.); adshoaibi@kfu.edu.sa (A.A.A.-S.); 2 Department of Biological Sciences, College of Science, King Faisal University, P.O. Box 400, Al-Ahsa 31982, Saudi Arabia; bchellapan@kfu.edu.sa; 3 Department of Arid Land Agriculture, College of Agricultural and Food Sciences, King Faisal University, P.O. Box 400, Al-Ahsa 31982, Saudi Arabia; salganainy@kfu.edu.sa (S.M.E.); malmghaslah@kfu.edu.sa (M.I.A.); 4 Department of Physics, College of Science, King Faisal University, P.O. Box 400, Al-Ahsa 31982, Saudi Arabia

**Keywords:** tomato leaf curl Palampur virus, genetic diversity, population structure, phylogeography, spatiotemporal evolution, cross-border spread

## Abstract

Tomato leaf curl Palampur virus (ToLCPalV) is an economically important bipartite begomovirus in the agro-ecological regions in south and western Asia. This study was designed to investigate the sequence variation dynamics, regional delineation, genetic diversity, population structure, and cross-border dispersal patterns of ToLCPalV. The research revealed clear geographical structuring, with distinct Indo–Pak subcontinent and Middle Eastern clades, but no host-specific differentiation. Genetic diversity analysis indicated higher diversity in the Indo–Pak subcontinent, particularly in the DNA-B component, suggesting an older, more diverse population of ToLCPalV prevailing in this region. Neutrality tests and selection pressure analyses revealed predominantly purifying selection, with limited positive selection observed in BV1 of DNA-B. The primary source of dispersal of ToLCPalV progenitor was estimated in Varnasi, India in 1955, from where the virus was spread. No recombination events were detected, suggesting that mutation and selection are the primary drivers of ToLCPalV evolution. Furthermore, a detailed SDT-based nucleotide sequence comparison analysis also identified two potential strains of ToLCPalV. This study elucidates the spatiotemporal dynamics and evolutionary history of ToLCPalV, revealing its cross-border spread and adaptive evolution. These findings contribute to a more comprehensive understanding of begomovirus epidemiology and provide valuable insights into ToLCPalV’s phylogeography and evolutionary dynamics.

## 1. Introduction

Plant viruses significantly challenge worldwide agriculture, leading to diseases that can drastically reduce crop production. Among these, the tomato leaf curl Palampur virus (ToLCPalV) stands out as a key member of the *Begomovirus* genus in the *Geminiviridae* family, affecting numerous plant species across diverse agro-ecological regions in Asia [1]. ToLCPalV is classified as a bipartite begomovirus belonging to the Old World (OW) clade, comprising two single-stranded DNA genomic segments, each measuring approximately 2.6–2.7 kb in length, and encapsulated within a distinctive ‘geminate’ particle [2]. The transmission of ToLCPalV is primarily mediated by whiteflies (*Bemisia tabaci*), making it a highly efficient plant pathogen with substantial agricultural impacts. The *B. tabaci* species complex, part of the *Aleyrodidae* family, exhibits remarkable global genetic diversity, with an estimated 1500 cryptic species identified [3].

The development and dissemination of novel begomovirus strains, as well as their movement into previously unaffected areas, are driven by multiple factors. These include the global spread of the whitefly vector, the widespread presence of wild host plants, human activities, and viral evolution through recombination and pseudo-recombination [4,5]. Climate change also plays a significant role by altering the distribution patterns of insect vectors and their associated viruses, further complicating plant-virus interactions. The rapid adaptability of begomoviruses to infect new hosts exacerbates challenges in crop management and disease control [5]. Together, these factors shape the evolving distribution of begomoviruses, creating significant obstacles for agricultural management and disease mitigation. Native begomovirus species within a specific region may display genetic variations when compared to non-native species, yet they can undergo evolution via similar processes or through adaptations tailored to local conditions [6].

ToLCPalV, first identified in India in 2008, affects tomato crops [2]. It has since spread to various regions. In India, it has been observed infecting multiple vegetable crops, their wild relatives, and weeds [7,8,9,10]. The virus has also been reported in Pakistan, where it affects chili [11], pepper, papaya, mako [12], cucumber [13], bitter gourd [14], and melon. In Iran, ToLCPalV has been detected in tomato, muskmelon, cucumber, common bean, squash, and watermelon [15,16,17]. Additionally, the virus has been found in Iraq [18,19,20], Saudi Arabia [1,21], and Oman [22,23], infecting crops such as squash, cucumber, melon, tomato, and papaya. This widespread distribution highlights the potential for long-distance spread of begomoviruses.

In recent years, the intercontinental movement of viruses has become increasingly evident. For instance, the westward dissemination of tomato yellow leaf curl virus (TYLCV) across Africa in the 1990s, as documented by Mabvakure et al. [24], and the introduction of the northwestern squash leaf curl virus (SqLCV) into the Middle East, reported by Sobh et al. [25], illustrate this trend. Furthermore, the spread of cotton leaf curl disease (CLCuD) from the Indo–Pakistan region to China [26] and the more recent cross-continental migration of OW watermelon chlorotic stunt virus (WmCSV) to Mexico and the United States [27,28] further demonstrate the ability of viruses to traverse continents. Despite being identified relatively recently, ToLCPalV has rapidly expanded its host range, indicating a significant capacity for adaptation. However, the genetic diversity and evolutionary processes driving this adaptability remain poorly understood.

Geminivirus evolution and host adaptation are driven by a multifaceted interplay of factors, including the availability of alternative host species, genetic recombination, component acquisition, and selective pressures from host plants [29,30,31]. These elements significantly contribute to the genomic diversity of these viruses, facilitating their evolutionary success across diverse hosts and geographic locations [32,33,34,35]. The genetic variability observed in geminiviruses is substantial, comparable to that of RNA viruses, and is primarily driven by mutation and recombination [30]. This high degree of variability allows geminiviruses to adapt swiftly to changing environments, which is essential for their survival and widespread dissemination.

Numerous studies have examined ToLCPalV, yet its population history, evolutionary trajectory, and migration patterns within a temporal context remain inadequately understood. This study focuses on analyzing the temporal dynamics, phylogeography, and demographic history of ToLCPalV by utilizing all available sequences from GenBank. A primary objective is to identify patterns of geographical dispersion and trace the virus’s movement across two distinct populations: South Asia (India, Pakistan) and West Asia (Iran, Iraq, Oman, Saudi Arabia). The research aims to uncover the evolutionary, ecological, and anthropogenic factors influencing these populations, reconstruct evolutionary lineages to determine ancestral relationships among viral strains, and evaluate the role of selection pressure by identifying regions of the viral genome subject to positive or negative selection. Additionally, the study investigates other genetic diversity indices (GDIs) within the two ToLCPalV populations, extending the analysis beyond selection pressure alone.

## 2. Materials and Methods

### 2.1. Sequence Retrieval and Multiple Sequence Alignment

The full-length genomic sequences of ToLCPalV DNA-A (82 sequences) and DNA-B (54 sequences) were obtained from the NCBI GenBank database (https://www.ncbi.nlm.nih.gov/, accessed on 15 January 2025) (Table S1). Individual open reading frames (ORFs) were extracted from DNA-A (AV1, AV2, AC1, AC2, AC3, and AC4) and DNA-B (BV1 and BC1), forming eight subsets. Genetic diversity and population structure were evaluated by categorizing the ToLCPalV sequences into three distinct populations, namely Pop-1 (entire population of ToLCPalV), Pop-2 (ToLCPalV isolates from the Indo–Pak sub-continent), and Pop-3 (ToLCPalV isolates from the Middle East). A total of 30 datasets were generated, covering the complete genomes and individual ORFs of DNA-A and DNA-B components for each population. Multiple sequence alignments (MSAs) were performed using MEGA11 software employing the ClustalW algorithm [36]. To ensure accuracy, all alignments were manually inspected and corrected.

In accordance with established geminivirus classification guidelines, the species demarcation tool (SDTv1.2) was utilized to calculate pairwise nucleotide sequence identities [37] (Figure S1A,B).

### 2.2. Phylogenetic Analysis and Evolutionary Relationship Modeling of ToLCPalV

To assess the temporal signal strength within the dataset, we performed TempEst analysis using BEAST v.2.7.4. Subsequently, a root-to-tip plot was generated to visualize the regression of genetic distances against sampling time (Figure S2), offering insights into evolutionary timelines and supporting subsequent phylogenetic analyses.

**Figure 1:**
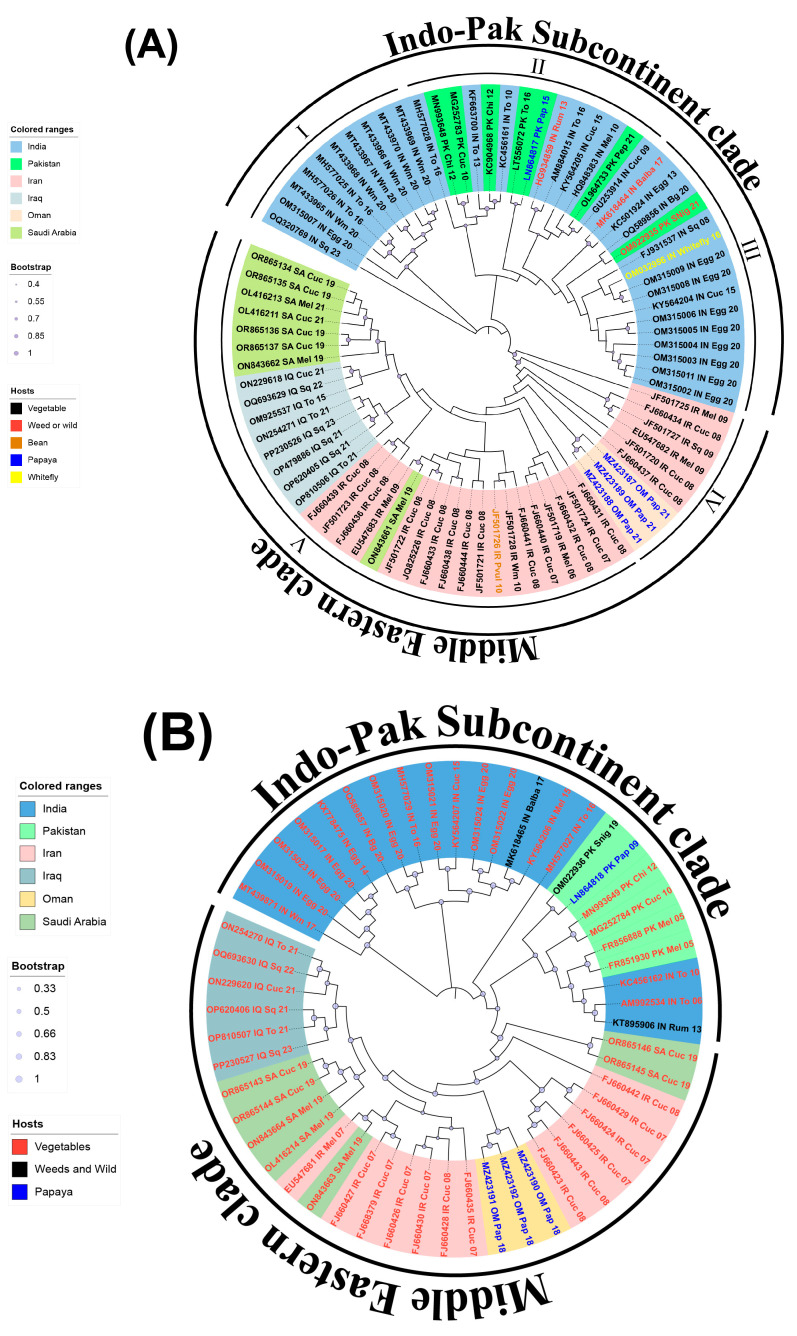
Maximum likelihood (ML) phylogenetic tree of tomato leaf curl Palampur virus (ToLCPalV) DNA-A (**A**) and DNA-B (**B**) full-length genomic sequences. The evolutionary history was inferred using the ML method in MEGA11, with 1000 bootstrap replicates for branch support. Colored ranges indicate different geographical origins, including India, Pakistan, Iran, Iraq, Oman, and Saudi Arabia, while different host types (vegetables, weeds/wild plants, beans, papaya, and whitefly) are represented by distinct markers. Bootstrap support values are indicated by circle size, with higher values reflecting greater confidence in clade formation. The resulting tree was visualized and edited using iTOL v6.5 and Adobe Illustrator CC.

For phylogenetic relationship inference, we analyzed the full-length DNA-A and DNA-B genomic components of ToLCPalV using MEGA11 software. Evolutionary distances were calculated using the maximum likelihood (ML) method, with 1000 bootstrap replicates to ensure robustness. The Tamura-Nei (TN93) model, incorporating gamma distribution and invariant sites (G+I), was selected as the best-fit nucleotide substitution model to address site heterogeneity. The resulting phylogenies were visualized and refined using graphical simulations in iTOL v6.5 (https://itol.embl.de/#, accessed on 4 January 2025) [38] and Adobe Illustrator CC (version 29.5) (Figure 1A,B). Time-scaled phylogenetic analysis was performed using a Bayesian approach in BEAST v.2.4 [39]. Parameters such as tree priors, molecular clock models, and demographic models were configured in BEAUti2 to create a BEAST control file. Phylogenetic inference was performed using BEAST v.2.7.4, with the Bayesian Markov Chain Carlo (MCMC) method optimized to achieve high probability density (HPD) intervals. Convergence and effective sample sizes (ESSs) were assessed using Tracer v.1.7.2 to ensure reliable posterior distributions. The final maximum clade credibility (MCC) tree was produced using TreeAnnotator v1.10.5 [40] and visualized in FigTree v1.4.4 (http://tree.bio.ed.ac.uk/software/figtree/, accessed on 1 February 2025). SpreaD3 v 0.9.6 software [41] was employed to analyze and visualize the temporal dynamics of ToLCPalV evolution.

### 2.3. Median-Joining Network Analysis of ToLCPalV

The median-joining (MJ) network was inferred using the aligned full-length DNA-A dataset of 82 ToLCPalV isolates in popART v.1.7 [42]. The MJ networks were constructed using popART v.1.7 based on the geographical and host distribution of the isolates, with input files generated using DnaSP.

### 2.4. Genetic Diversity and Neutrality Tests

Nucleotide diversity (π), which measures the average number of nucleotide differences per site between sequences, was calculated for all datasets using DnaSP v.6.12 [43], as described earlier [44]. The statistical significance of variations in π was determined by comparing 95% bootstrap confidence intervals. A sliding window analysis, with a window size of 100 nucleotides and a step size of 10 nucleotides, was conducted across the complete length of the viral genome sequences to evaluate genetic variation.

To assess the selection pressure acting on ToLCPalV populations and individual ORFs, several population genetic parameters were estimated for all datasets. These included the number of haplotypes (H), haplotype diversity (Hd), the number of polymorphic/segregating sites (S), Watterson’s theta (θw), average number of nucleotide differences between sequences (k), the total number of mutations (Eta), Tajima’s D (TD), and Fu and Li’s D (FLD).

### 2.5. Bayesian Analysis of ToLCPalV Evolution

The nucleotide substitution rate per site per year (NSSY) and mutation rates were calculated for the complete ToLCPalV dataset (Pop-1), its subpopulations (Pop-2 and Pop-3), and each individual ORF within these populations utilizing a Bayesian MCMC methodology executed in BEAST (v.1.10.5) with a chain length of 1 × 10^7^ [40]. To assess evolutionary dynamics, both strict and relaxed (uncorrelated lognormal) molecular clock models were applied for all ToLCPalV datasets. The BEAST-generated data were subsequently processed using Tracer software v.1.7.2 to identify the most suitable clock model, examine mutation rates across codon positions within ORFs, and confirm that ESS exceeded 200 for reliability.

### 2.6. dN/dS Ratio and FUBAR/SLAC Analyses for ToLCPalV

Two methods were employed to assess selection pressure acting on the ORFs of ToLCPalV. The first approach estimated the ratio of non-synonymous (dN) to synonymous (dS) substitutions using MEGA11 software. This ratio provides insights into the selection pressure, with a dN/dS ratio greater than 1 suggesting positive selection, a ratio less than 1 indicating negative selection, and a ratio close to 1 signifying neutral selection. Secondly, the Datamonkey server (www.datamonkey.org), was utilized to analyze the sequences using Fast, Unconstrained Bayesian AppRoximation (FUBAR) and Single-Likelihood Ancestor Counting (SLAC) methods. These methods offer alternative approaches to estimate selection pressure on the ORFs.

### 2.7. Determination of Putative Recombination Hotspots

This research employed a dataset consisting of 150 full-length DNA-A sequences and 100 full-length DNA-B sequences of begomoviruses. To identify potential parental sequences, only sequences with >70% nucleotide identity to the ToLCPalV genome were included in the recombination analysis, following the recommendation of Crespo-Bellido et al. [45]. Sequence alignments were performed using MEGA11, and the resulting FASTA files were analyzed for recombination events using GARD and the Recombination Detection Program (RDP v5.0) [46]. In RDP v5.0, seven different recombination detection algorithms were employed, and only events and breakpoints confirmed by at least three algorithms were retained. The analysis was conducted under default parameters, incorporating a Bonferroni-corrected *p*-value threshold of 0.05.

## 3. Results

### 3.1. Sequence Comparisons and Measuring Pairwise Nucleotide Sequence Identities

Based on SDT analysis, and adhering to ICTV guidelines with species demarcation set at 91% and strain demarcation at 94%, full-length genomic sequences of ToLCPalV DNA-A (82 isolates) and DNA-B (52 isolates) were compared (Figure S1A,B). The DNA-A sequences exhibited distinct clustering patterns corresponding to their geographical origin. For example, DNA-A isolates from the Middle East showed high mutual sequence identities, exceeding the 94% strain demarcation threshold. This suggests a relatively recent common ancestor for Middle Eastern isolates and active gene flow within this group.

In contrast, isolates from the Indo–Pak subcontinent displayed greater sequence divergence, both amongst themselves and compared to the Middle Eastern cluster. While some Indo–Pak subcontinent isolates shared similarities reaching 100%, many others fell below the 94% strain demarcation (Figure S1A). Specifically, the isolates MH577025 and MH577026 reported in tomatoes in India showed the highest nucleotide sequence identities of 93.7% and 93.9%, respectively, with a recently reported ToLCPalV isolate (OQ320769) from India [47]. Similarly, ToLCPalV isolates MT433966-70, identified in watermelons in India, shared the highest nucleotide sequence identities of 91.7–93.3% with ToLCPalV isolates AM884015, reported in tomatoes [2], and OM315007, reported in eggplants in India. A similar pattern was observed in their corresponding DNA-B isolates. The ToLCPalV DNA-B isolate MH577027 showed the highest nucleotide sequence identity of 91.7% with the isolate (KC456162) reported in tomatoes in India [48]. Meanwhile, the ToLCPalV DNA-B isolate MT439871 showed the highest nucleotide sequence identity of 86.6% with the isolate OM315023 infecting eggplants in India (Figure S1B).

Following ICTV recommendations, isolates MH577025 and MH577026 could be classified as belonging to a new strain. Isolates MT433966-70 tentatively form another distinct strain of ToLCPalV. These results indicate more substantial evolutionary distances within the Indo–Pak subcontinent group, suggesting a more complex evolutionary history potentially involving independent diversification and limited gene flow with the Middle Eastern group.

### 3.2. Phylogeography and Population Structure of ToLCPalV

**Figure 2:**
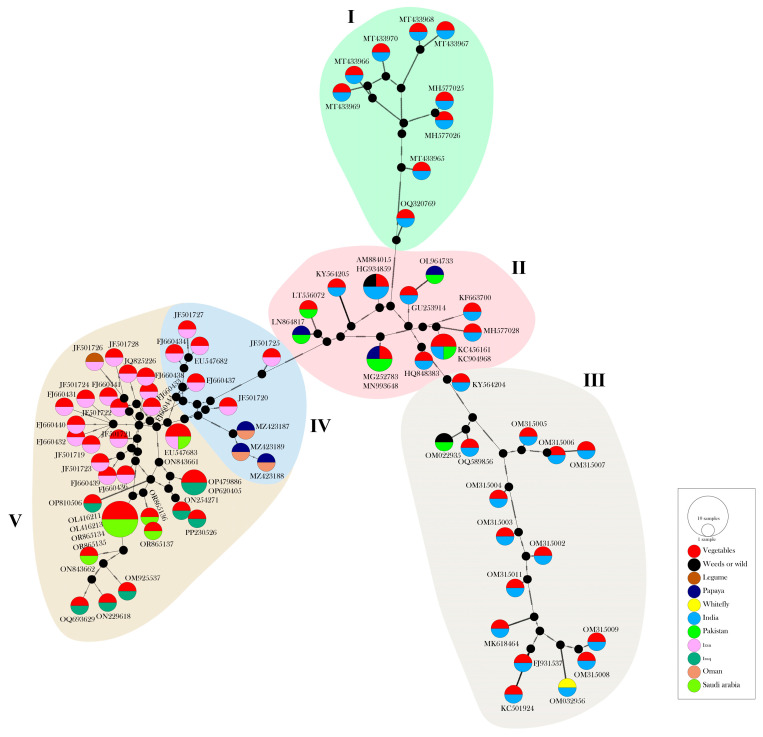
Median-joining network of tomato leaf curl Palampur virus (ToLCPalV) based on full-length DNA-A sequences constructed in PopART v1.7. Each colored circle represents a unique haplotype, with the size of the circle proportional to the number of isolates sharing that haplotype. Black dots indicate the missing ancestor sequences of ToLCPalV inferred by the algorithm. Pie charts within each circle illustrate the host distribution of each haplotype (vegetables, weeds/wild plants, legumes, whitefly, and papaya) and geographic origins (India, Pakistan, Iran, Iraq, Oman, and Saudi Arabia). The five major clusters (I–V) are highlighted with colored backgrounds corresponding to different regional clades. The network structure provides a visual representation of genetic relationships and geographical distribution patterns of ToLCPalV across different hosts and locations.

Phylogenetic analysis divided the 82 ToLCPalV DNA-A isolates into two major clades comprising the isolates identified from the Indo–Pak subcontinent, including India and Pakistan (the Indo–Pak subcontinent clade) and the isolates from Iran, Iraq, Oman, and Saudi Arabia (the Middle Eastern clade). While the isolates exhibited clear regional distinctions, no host-specific delineations were apparent (Figure 1A,B). The MJ network analysis also supported the ToLCPalV phylogeographic analysis, revealing distinct regional clustering without any clear differentiation based on host specificity (Figure 2). The Indo–Pak subcontinent clade encompassed 39 isolates (32 from India and 7 from Pakistan) originating from vegetables, papayas, weeds/wild plants, and whiteflies. These isolates were further classified into three sub-clades. Sub-clade I included 10 isolates from India, all infecting vegetables. Sub-clade II comprised 14 isolates (8 from India and 6 from Pakistan), from vegetables, weeds or wild plants, and papayas. Sub-clade III contained 15 isolates (14 from India and 1 from Pakistan) infecting vegetables, weeds or wild host plants, and whiteflies. Notably, the ToLCPalV isolate (OQ320769) from India clustered monophyletically in the sub-clade I in both analyses. The Middle Eastern clade was sub-divided into two sub-clades. Sub-clade IV encompassed nine ToLCPalV isolates (six from Iran and three from Oman) infecting vegetables and papayas. Sub-clade V comprises 34 isolates (18 from Iran, 8 from Iraq and 8 from Saudi Arabia). These isolates were detected in vegetables, legumes, and papaya. Interestingly, three ToLCPalV isolates (JF501725, JF501720, and FJ660437) formed distinct, monophyletic lineages in sub-clade IV within the phylogenetic and MJ analyses. In MJ network analysis, five distinct population groups (I–V) were identified, encompassing isolates from diverse geographical origins without explicit host-specific designations. Groups I–III comprised isolates predominantly from India and Pakistan, exhibiting regional delineation. Groups IV and V encompassed isolates predominantly found in Middle Eastern countries such as Iran, Iraq, Oman, and Saudi Arabia. Notably, some identical sequences were merged, and certain accessions were displayed as representatives. Within Group III, identical sequences were observed, such as MG252783 with MN993648, AM884015 with HG934859, and KC456161 with KC904968. Similarly, within Group V, identical sequences included EU547683 with ON843661, OP479886 with OP620405, and OL416211 with OR865134-OR865134, OL416213. Intriguingly, Groups IV and V, encompassing isolates from the Middle East, exhibited a significantly higher number of mutations compared to Groups I–III, which predominantly included isolates from the Indo–Pak subcontinent.

Similarly, the 54 DNA-B isolates clustered into two major groups, aligning with the Indo–Pak subcontinent clade (24 isolates: 18 from India and 6 from Pakistan) and the Middle Eastern clade (30 isolates: 13 from Iran, 6 from Iraq, 7 from Saudi Arabia, and 3 from Oman). This clustering mirrors the findings observed in the phylogenetic and MJ analyses for DNA-A, demonstrating clear regional demarcation without any apparent host-specific clustering within DNA-B (Figure 1B).

While no host-specific demarcation was observed for ToLCPalV, cucumber exhibited the widest distribution among vegetable hosts, with infections reported in five countries (India, Pakistan, Iran, Iraq, and Saudi Arabia). Melons were infected in four countries (India, Iran, Iraq, and Saudi Arabia), tomatoes in three (India, Pakistan, and Iraq), squash in three (India, Iran, and Iraq), papayas in two (Pakistan and Oman), and watermelons in two (India and Iran).

### 3.3. Comparative Analysis of Genetic Diversity and Neutrality Indices Across ToLCPalV Populations

The genetic diversity and neutrality indices across three ToLCPalV populations: the entire population of ToLCPalV (Pop-1), ToLCPalV isolates from the Indo–Pak sub-continent (Pop-2), and ToLCPalV isolates from the Middle East (Pop-3). The analysis also evaluated the respective ORFs within each population (Tables S2–S4). The key parameters included the InDel sites, S, Eta, Hd, π, k, θw, and neutrality test values (TD and FLD).

#### 3.3.1. Genetic Diversity Comparison

Comparative analysis of genetic diversity across three ToLCPalV populations revealed distinct patterns of variation. InDel site frequency was highest in the DNA-B of Pop-1 and Pop-2 (71 and 65 sites, respectively) and lowest in Pop-3 DNA-A (2 sites). Pop-1 DNA-B also exhibited the highest number of segregating sites (S = 1179) and total mutations (Eta = 1601), followed by DNA-B of Pop-2 (901 and 1115, respectively). Conversely, Pop-3 DNA-A had the lowest number of segregating sites (S = 361) and total mutations (Eta = 399). This pattern was mirrored in the number of variants, with DNA-B of Pop-1 showing the highest (556/258/67) and DNA-A of Pop-3 the lowest (98/14/2). While DNA-A of Pop-2 showed the highest haplotype diversity (Hd = 1), its DNA-B displayed the highest average nucleotide diversity (π = 0.09), mean pairwise nucleotide differences (k = 231), and Watterson’s theta (θw = 298.58). Conversely, Pop-3 exhibited the lowest values for all GDIs in both DNA-A and DNA-B (Table S4).

Among the ORFs datasets, the AV1 of Pop-2 showed the highest values for S (671), Eta (981), π (0.13), k (96.52), and θw (232.03), while the AC1 of Pop-1 had the highest h value (68). InDel sites were most frequent in the BC1 of Pop-2. Notably, the Hd was relatively consistent across ORF datasets, except for the AC4 of Pop-3, which had the lowest value (0.55). Interestingly, InDels were exclusively found in the AC4 (7) and AV1 (2) of Pop-3, while in the rest of the ORFs, no InDels were found. Pop-3 generally exhibited the lowest values for all other diversity indices across its ORFs. The k estimation was the highest in Pop-2 and the lowest in Pop-3 across all datasets. Haplotype diversity remained high (~0.99) across all three populations, except for AC4 of Pop-3, which exhibited the lowest value (0.55).

Overall, Pop-1 harbored the highest genetic diversity (Table S2), particularly in DNA-B, suggesting an older, more diverse population. Pop-2 exhibited moderate genetic variation but the highest nucleotide diversity, indicating active genetic differentiation (Table S3). Pop-3 displayed the lowest genetic diversity and the strongest purifying selection (Table S4), possibly due to a recent bottleneck or selective sweep.

**Figure 3:**
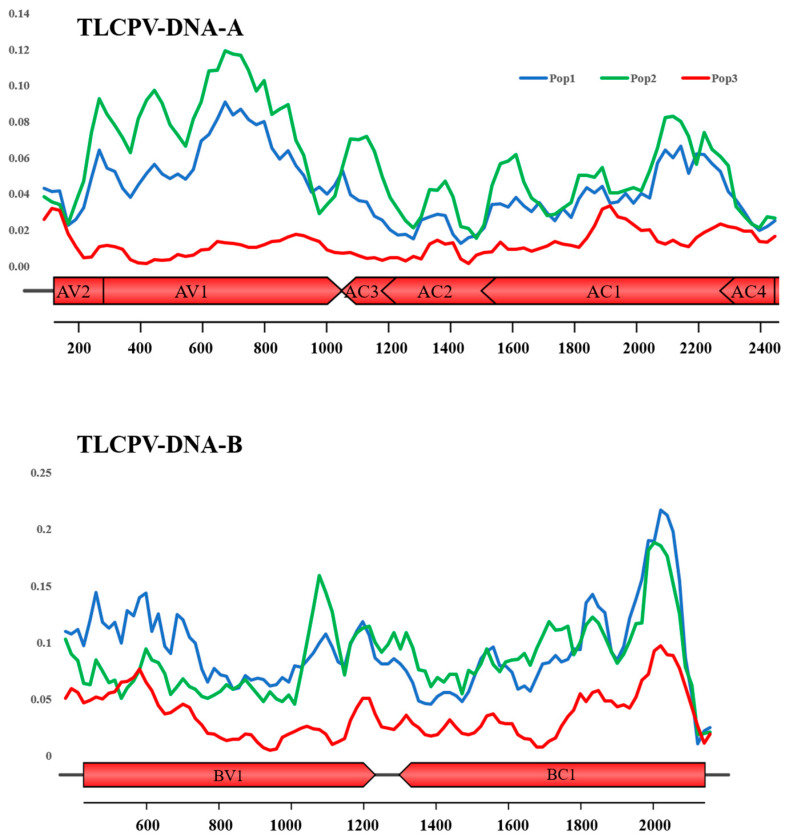
Nucleotide diversity of ToLCPalV DNA-A and DNA-B components. The figure illustrates the nucleotide diversity (π) across the complete genome sequences of the tomato leaf curl Palampur virus (ToLCPalV), DNA-A (top panel), and DNA-B (bottom panel) for three distinct populations: Pop-1 (blue line), Pop-2 (green line), and Pop-3 (red line). The x-axis represents the nucleotide position along the viral genome. The approximate locations of open reading frames (ORFs) AV2, AV1, AC3, AC2, AC1, AC4 (on DNA-A) and BV1, BC1 (on DNA-B) are indicated by red boxes below the x-axis. The y-axis represents the nucleotide diversity (π) at each position, calculated using a sliding window approach. Distinct colors represent the three ToLCPalV populations: Pop-1 (blue), Pop-2 (green), and Pop-3 (red).

The nucleotide diversity was analyzed across all nucleotide positions in the DNA-A and DNA-B components for the three ToLCPalV populations (Pop-1, Pop-2, and Pop-3) (Figure 3). In DNA-A, Pop-2 exhibits the highest nucleotide diversity across most regions, with peaks exceeding 0.12 at multiple positions. Pop-1 follows a similar pattern but with generally lower nucleotide diversity, showing peaks around 0.1. Pop-3 consistently displays the lowest nucleotide diversity, remaining below 0.05 throughout. Notable regions with increased nucleotide diversity include AV1, AC3, AC2, and the N-terminal region of AC1, particularly in Pop-1 and Pop-2. In DNA-B, the highest nucleotide diversity is observed in the BC1 region, particularly in Pop-1 and Pop-2, where diversity surpasses 0.2 in some positions. Pop-2 again shows the highest diversity, followed by Pop-1, while Pop-3 remains the least diverse. The BV1 region also exhibits moderate nucleotide diversity, with Pop-2 and Pop-1 showing more variation than Pop-3. Overall, Pop-2 has the highest nucleotide diversity across both DNA-A and DNA-B, followed by Pop-1, while Pop-3 remains the most conserved.

#### 3.3.2. Neutrality Indices Comparison

Neutrality tests (TD and FLD) were negative across all ToLCPalV populations (Tables S2–S4). The highest negative TD (−2.32) and FLD (−4.20) values were calculated for the DNA-A of Pop-3. In DNA-B, Pop-3 had the most negative TD (−1.44), while Pop-1 had the most negative FLD (−1.04) values. Interestingly, the DNA-B of Pop-2 had the least negative TD (−0.92) and FLD (−0.96) values, indicating the limited role of natural selection in the diversification of Pop-2.

Among ORF datasets, the AC4 of Pop-3 had the most negative TD (−2.66) and FLD (−5.31) values. The least negative TD (−0.12) was observed in the AV2 of Pop-2, and the least negative FLD (−0.19) in the BV1 of Pop-3. The BC1 datasets across all three populations showed notably low TD and FLD values.

### 3.4. Estimation of Evolutionary Rate

#### 3.4.1. Mean Substitution Rates

Overall, the mean NSSY rates for DNA-A in Pop-2 (8.23 × 10^−4^) and Pop-1 (7.63 × 10^−4^) were slightly higher than in Pop-3 (7.06 × 10^−4^) under the strict molecular clock, indicating only minor variations between these populations (Tables S5 and S6). However, for DNA-B, Pop-3 exhibited the highest overall NSSY rate, at 8.81 × 10^−4^ (95% HPD ranging between 7.250 × 10^−4^ to 1.048 × 10^−3^) under strict clock and 9.40 × 10^−4^ (95% HPD ranging between 7.801 × 10^−4^ to 1.112 × 10^−3^) under relaxed clock (Table S7), suggesting a greater evolutionary rate compared to both Pop-1 and Pop-2.

Analysis of DNA-A ORFs revealed varying NSSY rates across different populations. For AC1, the strict model showed the highest rate in Pop-2 (1.04 × 10^−3^) and the lowest in Pop-1. However, the relaxed model indicated a significantly lower rate for Pop-2 (3.77 × 10^−4^), suggesting more variable evolutionary dynamics. For AC2, Pop-1 exhibited a slightly higher strict mean rate (3.25 × 10^−4^) compared to Pop-2 (2.78 × 10^−4^) and Pop-3 (2.97 × 10^−4^), a trend that continued under relaxed models, indicating a more stable evolutionary rate. AC3 showed similar rates for Pop-1 and Pop-2 (~4.32 × 10^−4^ and 3.74 × 10^−4^, respectively), with Pop-3 having the lowest rate (2.73 × 10^−4^ under the strict model). The relaxed model for Pop-1 was the highest among the three populations. Pop-3 had a higher AC4 substitution rate (4.75 × 10^−4^) than Pop-1 (3.05 × 10^−4^) and Pop-2 (2.10 × 10^−4^), suggesting stronger selective pressures or genetic drift. AV1 showed the highest rate in Pop-2 (6.81 × 10^−4^), while AV2 exhibited similar rates in Pop-1 and Pop-2 (~2.59 × 10^−4^ and 2.40 × 10^−4^, respectively), with a slightly lower rate in Pop-3 (2.10 × 10^−4^), indicating a slower evolutionary rate in this population.

For DNA-B ORFs, BV1 in Pop-3 demonstrates the highest substitution rate (5.31 × 10^−4^) compared to Pop-1 (4.53 × 10^−4^) and Pop-2 (3.80 × 10^−4^), a trend mirrored in the relaxed model. Similarly, for BC1, Pop-3 exhibits the highest rate under both strict (5.61 × 10^−4^) and relaxed (5.92 × 10^−4^) models, suggesting greater evolutionary pressure on this ORF.

#### 3.4.2. Codon Position Mutation Rates

Analysis of codon position mutation rates revealed varying selective pressures across the three populations (Tables S8–S10). For the first codon position (CoP1), Pop-1 showed the highest mutation rates in AC2 (1.127) and BV1 (1.762), indicating strong selection. Pop-2 generally exhibited lower CoP1 values, suggesting less variation at this position, while Pop-3 presented mixed values, with a high rate in BV1 (1.912) but lower rates in other ORFs. Regarding the second codon position (CoP2), Pop-1 displayed varied mutation rates, ranging from a low of 0.383 in AV1 to a high of 1.58 in AC4. Pop-2 had an overall lower CoP2 mutation rate, particularly in AC2 (0.941) and AC3 (0.884). Pop-3 generally showed lower CoP2 mutation rates, suggestive of strong selective constraints. For the third codon position (CoP3), Pop-1 exhibited high rates in AC3 (1.469) and AV1 (1.377), indicating increased substitution frequency. Pop-2 showed some of the highest rates in AV1 (2.05) and AV2 (1.512), indicating increased dS rates. Pop-3 generally had higher CoP3 values in BV1 (1.912) and BC1 (2.27), suggesting greater genetic variability. Finally, a comparison of strict and relaxed molecular clock models showed minor variation for Pop-1, except for AC1, where the relaxed model significantly increased the rate. Pop-2 exhibited substantial differences in AC1 and AC4, with the strict model yielding higher rates. Pop-3 showed similar values between the two models, suggesting more consistent evolutionary pressure.

A comparison of strict and relaxed molecular clock models revealed differing patterns across populations. Pop-1 showed only minor variations between the two models, except for AC1, where the relaxed model resulted in a significant increase in rate. In contrast, Pop-2 exhibited substantial differences in AC1 and AC4, with the strict models yielding higher rates than the relaxed models. Pop-3, however, showed similar values between the strict and relaxed models, suggesting more consistent evolutionary pressure.

### 3.5. Estimation of Selection Pressure

Selection pressure estimation on individual ORFs was assessed using mean genetic distance (d), FUBAR, SLAC, and dN/dS ratio (Tables S8–S10). Analysis of mean genetic distances revealed that Pop-2 exhibited the highest values, notably for AV1 (0.328) and AC4 (0.17), suggesting greater genetic variation within this population. Conversely, Pop-3 displayed the lowest mean distances, with values as low as 0.006 for AV2 and 0.008 for AC3, indicating a more conserved sequence set.

Analysis of selection pressure using dN/dS ratios revealed that most ORFs across all ToLCPalV populations were under purifying selection, indicating evolutionary constraints to maintain protein function. Notably, the ORFs in Pop-3 exhibited the lowest overall dN/dS ratios, suggesting stronger purifying selection compared to Pop-1 and Pop-2. Evidence of positive selection (dN/dS > 1) was observed only in BV1 for Pop-1 (1.299) and Pop-2 (1.343). Further analysis using FUBAR (*p* ≤ 0.9) showed that Pop-2 had a slightly higher number of positively selected sites (PS) compared to Pop-1 and Pop-3. The highest PS counts were five in AV2 (Pop-1 and Pop-2) and three in AC1 and AV1 (Pop-2). Pop-3 generally showed fewer PSs, indicating fewer adaptive changes. However, analysis using SLAC (*p* ≤ 0.05) detected no PS. Furthermore, the FUBAR and SLAC analyses detected the highest number of negatively selected sites (NS) in BC1 (123 and 58, respectively), followed by BV1 (121 and 45), AV1 (108 and 62), and AC1 (102 and 51) in Pop-1. Both FUBAR and SLAC analyses revealed that negative selection dominated all populations, with the highest counts in AV1, AC1, BV1, and BC1. Key differences among the populations included higher genetic diversity and selection pressure in Pop-1 and Pop-2, particularly in BV1 and BC1. In contrast, Pop-3 was the most conserved, with lower mean genetic distances, dN/dS ratios, and fewer PS/NS. Pop-2 exhibited the greatest variation in mean genetic distances and selection pressures across different ORFs.

### 3.6. Recombination Analysis

The recombination analysis conducted using two independent methods, i.e., RDP and GARD analysis, did not identify any potential recombination events in either the DNA-A or DNA-B genomic components of ToLCPalV. Thus, the genetic diversification of ToLCPalV is likely due to mutation and selection pressure rather than recombination.

### 3.7. The Spatiotemporal Evolution of ToLCPalV

**Figure 4:**
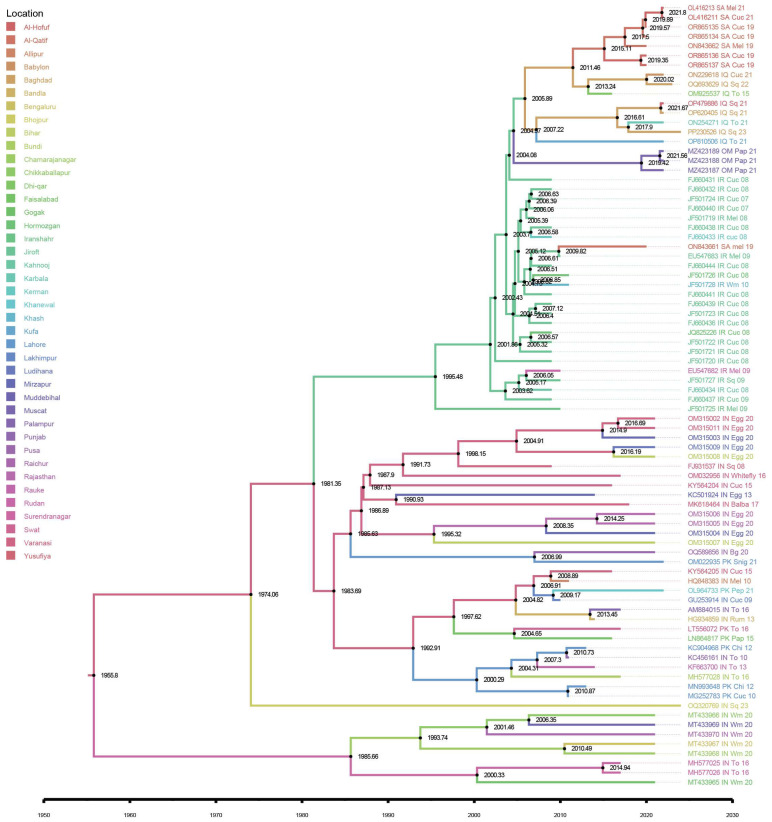
Representation of a time-scaled phylogenetic tree of 82 complete DNA-A sequences of the tomato leaf curl Palampur virus (ToLCPalV), reconstructed using a Bayesian Markov Chain Monte Carlo (MCMC) approach implemented in BEAST v.2.7.4, employing a relaxed molecular clock model and a coalescent Bayesian skyline demographic model. Node ages are indicated along the timeline axis, with estimated divergence times labeled at key nodes. Posterior probabilities are represented as branch support values. Geographic locations of virus isolates are color-coded, corresponding to their specific locations. The estimated time of origin of ToLCPalV is 1955. Branch lengths are proportional to the estimated time span of divergence. The branch tips were labeled using the accession number of the respective isolate, the country, host, and collection year. Visualization and annotation were performed using FigTree v1.4.4 and Adobe Illustrator CC.

The spatiotemporal spread of ToLCPalV was investigated by constructing a time-scaled phylogenetic tree using the complete genome sequences of 82 ToLCPalV DNA-A isolates. The tree was generated through the MCMC method implemented in BEAST. The MCC tree provided insights into the virus’s evolutionary history, divergence times, and geographical distribution. The findings from the MCC tree aligned with those of the ML phylogenetic tree and MJ network analysis, reinforcing the classification of ToLCPalV isolates into two primary groups: one originating from the Indo–Pak subcontinent and the other from the Middle East (Figure 4).

The estimated divergence times ranged from 1955 to recent years (2021 and beyond), indicating both long-term persistence and ongoing evolution. The earliest genetic divergence was inferred to have occurred in 1955, followed by major bifurcation events in 1974, 1981, and 1995. These events suggest early diversification of ancestral strains, with more recent sub-clades implying active recombination or mutation leading to newer variants. The non-linear progression of bifurcated clades underscores high genetic diversity within the ToLCPalV population. Geographic clustering of isolates from regions such as India, Pakistan, Iraq, Iran, and the Arabian Peninsula suggests localized transmission and adaptation, while close clustering of isolates from distinct countries indicates possible cross-border movement. Significant divergence was observed in isolates from Punjab, Faisalabad, Bihar, and Baghdad, indicating independent evolutionary trajectories. The clustering of several strains around the 2010s and 2020s suggests recent viral expansion or the emergence of new variants. The presence of closely related isolates across different locations highlights potential transmission through trade or vector movement.

**Figure 5:**
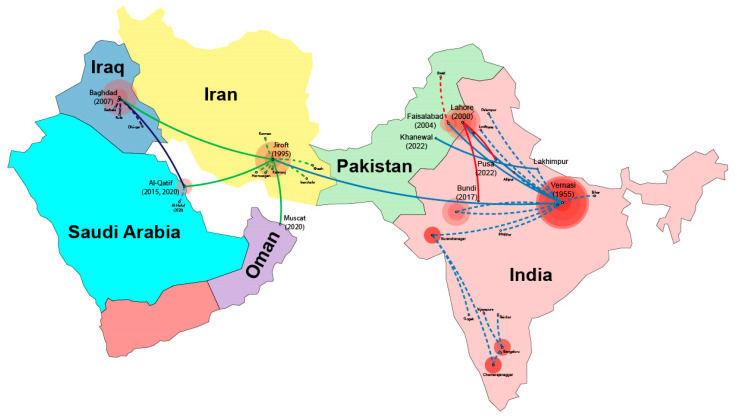
Spatiotemporal dispersal of the tomato leaf curl Palampur virus (ToLCPalV) DNA-A based on Bayesian phylogenetic and phylogeographic analyses. The map illustrates the inferred origin and geographic spread of ToLCPalV DNA-A over time. Red circles indicate the estimated viral origin and subsequent dispersal events, with the size of each circle proportional to the relative viral population size at that location. Solid lines represent inferred international transmission routes, with arrows denoting the direction of viral spread. Dispersal from India is shown in blue, from Pakistan in red, from Iran in green, and from Iraq in purple. Dotted lines in the respective colors represent local transmission within each country. Visualization and annotation were performed using Adobe Illustrator CC.

Temporal signal validation through TempEST analysis estimated the evolutionary origin of the ToLCPalV progenitor around 1940 (Figure S2). Visualization of the MCC tree with Spread3 v 0.9.6 software further elucidated the spatiotemporal dynamics of ToLCPalV DNA-A dispersal. India serves as the primary origin of ToLCPalV, with the virus likely originating in Varanasi in 1955, where the virus remained localized until 1995 (Figure 5). From this initial point, early within-country spread (indicated by blue dotted lines) is evident, with transmission to regions such as Lakhimpur, Surendranagar, Chamarajanagar, and Bengaluru demonstrating continued spread through multiple southward and eastward movements. Subsequently, cross-border transmission from India (highlighted by blue continuous lines) first occurred in Pakistan, with notable movements through Lahore (2000) and Faisalabad (2004) via Varanasi. A second introduction route extended from Lakhimpur, India, into Khanewal, Pakistan (2022). Pakistan acted as a secondary hub for dissemination, with the virus spreading from Lahore, Pakistan, back to Bundi (2017) and Pusa (2022) in India, indicated by red continuous lines, and further within-country transmission in Pakistan from Faisalabad to Swat (red dotted lines).

An early westward virus expansion was also noted, originating from Varanasi, India, and reaching Jirof, Iran, by 1995 (Figure 5). Iran played a significant role in the regional dissemination of ToLCPalV in the Middle East, with virus movement from Jiroft (1995) to Iraq (Baghdad, 2007) shown by green continuous lines, and further westward spread towards the Arabian Peninsula, reaching Al-Qatif, Saudi Arabia (2015), and Muscat, Oman (2020). In Saudi Arabia, ToLCPalV appears to have been introduced twice into Al-Qatif: once from Jiroft, Iran, in 2015, and again from Baghdad, Iraq, in 2020. The presence of ToLCPalV in Al-Qatif and Muscat suggests ongoing viral expansion and possible adaptation to new environments. Spatial clustering of sequences suggests regional adaptation and lineage formation. Transmission pathways likely involve a combination of natural and human-mediated dispersal, potentially through infected plant material, vector movement, or trade routes. The observed recent virus movement within India and Pakistan (2022) highlights ongoing viral evolution and the emergence of new outbreaks. Overall, the MCC tree analysis reveals a complex transmission pattern, with ToLCPalV DNA-A originating in India and spreading westward. The virus continues to evolve, with recent diversification events in India and Pakistan underscoring the need for ongoing monitoring and control measures.

## 4. Discussion

As the largest genus of plant viruses, begomoviruses are responsible for substantial crop losses; however, their evolutionary history remains largely unclear. This research addresses this knowledge gap by applying phylodynamic methods to ToLCPalV, a critical pathogen in the Indo–Pak subcontinent and the Middle East. Begomoviruses are known for high recombination rates [30], which can hinder phylodynamic studies. However, the ToLCPalV genome was found to be free of recombination in this detailed study, making it an ideal candidate for this type of analysis. The abundance of full-length genomic sequences of begomoviruses in GenBank, a result of their relatively small genomes, provides a robust dataset for such an evolutionary study. Accurate cataloging of viral diversity necessitates strict adherence to ICTV guidelines. In this study, we identified two previously unrecognized ToLCPalV strains within published isolates. Specifically, isolates MH577025 and MH577026 represent one novel strain, while isolates MT433966-70 constitute another.

The study provides valuable insights into the genetic variability, population structure, and cross-border spread patterns of ToLCPalV. The higher genetic diversity within the Indo–Pak subcontinent, compared to the Middle East, points towards a longer period of independent evolution, potentially coupled with limited gene flow between the two regions. The phylogenetic analysis further revealed a clear regional differentiation of ToLCPalV populations, with distinct clades and sub-clades corresponding to South Asia (India and Pakistan) and West Asia (Iran, Iraq, Oman, and Saudi Arabia). This regional clustering, evident in both phylogenetic and MJ network analyses, suggests limited gene flow between these two major populations, likely due to geographical barriers and localized vector transmission dynamics. As highlighted by MacDonald et al. [49], such geographical or ecological isolation can lead to spatially divergent viral lineages. This pattern is consistent with findings in other begomoviruses, such as the tomato leaf curl New Delhi virus (ToLCNDV), where genetic distinctions are evident between isolates originating from the Indian subcontinent and those found in other geographical areas [50]. While host-specific clustering was not observed, the wide distribution of ToLCPalV across various crops, particularly cucumbers and melons, highlights its ability to infect diverse hosts and underscores the potential for cross-species transmission. Such regional delineation and absence of host-specificity have also been reported for the pedilanthus leaf curl virus (PeLCV) [51]. This lack of strict host specificity may contribute to the virus’s successful spread and adaptation to new environments, enhancing its evolutionary success. The ability of ToLCPalV to infect a wide range of hosts, including cucumber and melon, underscores its potential for cross-species transmission and successful adaptation to diverse environments. Notably, the co-occurrence of ToLCPalV and Zucchini yellow mosaic virus (ZYMV) in muskmelon within Pakistan suggests a complex interplay between different viruses and host plants [52]. Viral host range influences selective pressures, driving genetic diversity and adaptation. However, transmission efficiency in begomoviruses is also significantly impacted by different *B. tabaci* biotypes [53]. Notably, Asia II-1 efficiently transmits cotton leaf curl Multan virus in the Indo–Pak region compared to other biotypes [54]. The Indo–Pak region and the Middle East exhibit diverse *B. tabaci* populations. Specifically, MEAM1, Asia I, Asia II-1, and Asia II-5 are common in the Indo–Pak region [3]. In the Middle East, MEAM1 predominates in Saudi Arabia’s Eastern Province [55], MED in Fayfa [56], and both MEAM1 and Asia I variants are found in Iran [57]. Oman is dominated by MEAM1 [58], and Iraq reports both MEAM1 and MED [59]. These ecological settings may experience unique selection pressures, including host availability, environmental factors, and vector dynamics [60]. Over time, these pressures can result in the accumulation of genetic changes, driving the differentiation of viral populations within a particular environment [61].

The higher number of mutations observed in the Middle Eastern isolates of ToLCPalV compared to those from the Indo–Pak subcontinent suggests a potentially longer evolutionary history or more dynamic diversification in the West Asian population. Divergent vector populations, a wide array of host plant species, and regionally distinct selective forces may all play a role in generating these variations. The diverse *B. tabaci* biotypes present in both regions, such as MEAM1, Asia I, and Asia II variants, likely play a crucial role in ToLCPalV transmission and could contribute to the observed genetic differentiation. The B and Q biotypes of *B. tabaci*, for instance, have been shown to differ in their virus transmission efficiencies and insecticide resistance profiles, potentially affecting virus spread and evolution [62]. Investigating the specific roles of these biotypes in virus epidemiology remains crucial for understanding their contribution to ToLCPalV dispersal and adaptation and their potential influence on virus evolution.

Our analysis of GDIs revealed distinct patterns across the three ToLCPalV populations. Pop-1, representing the entire dataset, exhibited the highest overall genetic diversity. Most of the GDIs of Pop-2, comprising isolates from the Indo–Pak subcontinent, were comparable with Pop-1, particularly in DNA-B, suggesting a potentially older and more diverse ancestral population than Pop-3, encompassing Middle Eastern isolates. Pop-2 showed moderate genetic variation (InDels and Hd values) but the highest nucleotide diversity (π and θw), indicating active genetic differentiation within this region. In contrast, Pop-3 displayed the lowest genetic diversity and strongest signals of purifying selection, indicating a highly homogenous ToLCPalV population experiencing significant evolutionary constraints in the Middle East. These differences in genetic diversity and selection pressure may reflect distinct evolutionary trajectories shaped by varying environmental conditions, host pressures, and anthropogenic factors such as agricultural practices and trade-related virus dispersal.

Begomovirus evolution is significantly influenced by both point mutations and recombination, leading to genetic diversification [51,63]. Among the three ToLCPalV populations, Pop-2 exhibited a slightly higher NSSY for DNA-A (8.23 × 10^−4^). Notably, the DNA-B segment of Pop-3 exhibited the highest substitution rates, registering 8.81 × 10^−4^ and 9.40 × 10^−4^ under strict and relaxed molecular clock models, respectively. Although the NSSY rates were estimated with both strict and relaxed molecular clock models, the results from the strict clock were more promising. DNA viruses typically exhibit lower NSSY rates in the range of 10^−6^ to 10^−8^ as compared to RNA viruses, which generally exhibit higher rates in the range of 10^−3^ to 10^−5^. However, our results, consistent with previous studies, showed that geminivirus NSSY rates are comparable to those of RNA viruses. For instance, PaLCV [64], EACMV [65], ChiLCV [66] and TYLCV [34] exhibited NSSY rates between 10^−3^ and 10^−5^. The genetic structure of viral populations is under the direct influence of mutations, recombination, neutral selection, genetic drift, and gene flow [63]. The lack of any putative recombination event in our ToLCPalV genome analysis and the presence of a high NSSY rate indicate that point mutations are the dominant force in ToLCPalV evolution. This implies a distinct adaptation strategy and underscores the need for additional research. Codon position mutation rates suggest varying selection pressure among populations, with Pop-2 showing stronger selection at the first codon position, while Pop-3 had a greater mutation frequency at the third codon position. Overall, Pop-3 exhibited the highest evolutionary rates in DNA-B, while Pop-2 showed a higher rate in DNA-A. Thus, the three ToLCPalV populations experienced varying degrees of selection pressure across their codon positions and ORFs.

The selection pressure analysis using dN/dS ratios across ToLCPalV populations consistently revealed a dominant pattern of purifying selection, indicating strong evolutionary constraints on protein function. Positive selection (dN/dS > 1) was only observed in the BV1 gene for Pop-1 (1.299) and Pop-2 (1.343). BV1 encodes a protein involved in viral movement and replication [67]. Positive selection observed in this region may suggest adaptive changes linked to host interaction or vector-mediated transmission dynamics [29]. Further functional characterization of BV1 is necessary to elucidate the implications of these positively selected sites and their role in ToLCPalV adaptation and fitness. Pop-2 exhibited the highest mean genetic distances and the greatest variation in selection pressures, suggesting a more diverse and potentially adaptable population. Notably, Pop-3 emerged as the most conserved population, with the lowest mean genetic distances, dN/dS ratios, and fewer positively and negatively selected sites, suggesting stronger purifying selection and a highly conserved evolutionary trajectory. The FUBAR analysis indicated a slightly higher number of PS in Pop-2, particularly in AV2, AC1, and AV1, suggesting potential adaptive changes in these ORFs. However, this finding was not corroborated by the SLAC analysis, which detected no PS. This discrepancy highlights the potential sensitivity of different methods to detect positive selection and warrants cautious interpretation of the FUBAR results. However, both FUBAR and SLAC analyses consistently identified a significant number of NS across all populations, with BC1, BV1, AV1, and AC1 showing the highest counts. This confirms the strong influence of purifying selection in maintaining the functional integrity of these ORFs. This was further supported by highly negative TD and FLD values across all the populations and their encoded ORFs, signifying their conserved nature. The results suggest that most of the observable nucleotide variations in such viral populations are transient and eventually may be eliminated by purifying selection [66]. Such observations have been documented previously for different plant viruses [66,68]. Our detailed analysis implies that DNA-B might have a significant role in the evolutionary trajectory of ToLCPalV, particularly in the Middle East.

Spatiotemporal analysis of ToLCPalV dispersal, based on time-scaled phylogenetic trees, revealed a complex pattern of virus movement. First reported in 2008, ToLCPalV might seem like a recently emerged virus [2]; however, our study estimated that the most recent common ancestor of the sampled ToLCPalV isolates dates to approximately 1955. This suggests that the virus has been circulating and evolving for several decades prior to its formal identification. India appears to be the primary origin of ToLCPalV, with subsequent spread to Pakistan, Iran, and beyond. Both Pakistan and Iran acted as secondary dispersal hubs, facilitating further transmission within their respective regions and contributing to cross-border spread. The westward expansion of ToLCPalV from India to Iran and subsequently to the Arabian Peninsula highlights the potential for long-distance dispersal, likely mediated by a combination of natural factors (e.g., wind dispersal of whiteflies) and human-mediated activities (e.g., movement of infected plant material). These findings align with previous reports on the whitefly-mediated begomovirus cross-border dispersal. The recent emergence of new ToLCPalV variants observed in India, Pakistan, and the Middle East [1,11,12,21,47] underscore the ongoing evolution and spread of ToLCPalV. The increasing detection of bipartite begomoviruses in the Middle East [1,18,69] underscores the critical need for ongoing surveillance and robust viral disease management strategies [70].

This detailed phylodynamic analysis suggests that ToLCPalV is likely to continue its westward expansion from the Indo–Pak subcontinent into the Middle East, following established dispersal routes. Given the limited gene flow between these regions, the virus may further diverge into distinct regional lineages due to ecological isolation, vector biotype differences, and localized selection pressures. India may remain an evolutionary hotspot, serving as the major source of ToLCPalV variants, while Pakistan and Iran might act as secondary hubs, facilitating further virus movements into the Arabian Peninsula. Human-mediated transport of infected plant material and natural whitefly dispersal are likely drivers of long-distance spread. Proactive measures, such as resistant crop development, whitefly biocontrol, and international phytosanitary cooperation, could mitigate further dispersal of ToLCPalV.

## 5. Conclusions

This study provides a comprehensive overview of ToLCPalV’s genetic diversity, population structure, and dispersal patterns. However, some limitations should be acknowledged. The reliance on GenBank data, while providing a large dataset for analysis, may introduce biases due to uneven sampling across different regions and time periods. Future studies incorporating more comprehensive sampling and field-based data collection would provide a more complete picture of ToLCPalV dynamics. Additionally, further research is needed to investigate the specific roles of different *B. tabaci* biotypes in ToLCPalV transmission, to explore the functional significance of positively selected sites in BV1, and to assess the impact of environmental factors on virus evolution and spread. Despite these limitations, our study contributes significantly to our understanding of ToLCPalV evolution and provides a foundation for future research aimed at developing effective strategies for disease management and control.

## Data Availability

The raw data supporting the conclusions of this article will be made available by the authors on request.

## Supplementary Materials

The following supporting information can be downloaded at: https://www.mdpi.com/article/10.3390/v17050678/s1, Figure S1: Pairwise nucleotide sequence identities for tomato leaf curl Palampur virus (ToLCPalV) DNA-A and ToLCPalV DNA-B; Figure S2: Root-to-tip regression analysis of the full-length ToLCPalV DNA-A; Table S1: Complete dataset of ToLCPalV DNA-A and DNA-B isolates, including their associated plant host species, year of sample collection, and location of identification, along with corresponding geographical coordinates; Table S2: Estimation of genetic diversity and neutrality test indices for ToLCPalV DNA-A and DNA-B and their encoded ORFs in Pop-1 dataset; Table S3: Estimation of genetic diversity and neutrality test indices for ToLCPalV DNA-A and DNA-B and their encoded ORFs in Pop-2 dataset; Table S4: Estimation of genetic diversity and neutrality test indices for ToLCPalV DNA-A and DNA-B and their encoded ORFs in Pop-3 dataset; Table S5: Mean substitution and codon position mutation rate of the ToLCPalV Pop-1 and their encoded ORFs; Table S6: Mean substitution and codon position mutation rate of the ToLCPalV Pop-2 and their encoded ORFs; Table S7: Mean substitution and codon position mutation rate of the ToLCPalV Pop-3 and their encoded ORFs; Table S8: Estimation of selection pressure on the ORFs encoded by ToLCPalV isolates in Pop-1; Table S9: Estimation of selection pressure on the ORFs encoded by ToLCPalV isolates in Pop-2; Table S10: Estimation of selection pressure on the ORFs encoded by ToLCPalV isolates in Pop-3.

## Abbreviations

The following abbreviations are used in this manuscript:

ToLCPalV	Tomato leaf curl Palampur virus
OW	Old World
kb	Kilobase
*B. tabaci*	*Bemisia tabaci* (whitefly)
sp.	Species
TYLCV	Tomato yellow leaf curl virus
SqLCV	Northwestern squash leaf curl virus
CLCuD	Cotton leaf curl disease
WmCSV	Watermelon chlorotic stunt virus
GDIs	Genetic diversity indices
NCBI	National Center for Biotechnology Information
URL	Uniform Resource Locator
ORFs	Open reading frames
AV1	Coat protein gene (DNA-A)
AV2	Pre-coat protein gene (DNA-A)
AC1	Replication initiator protein gene (DNA-A)
AC2	Transcription activator protein gene (DNA-A)
AC3	Replication enhancer protein gene (DNA-A)
AC4	Gene involved in symptom development (DNA-A)
BV1	Movement protein gene (DNA-B)
BC1	Nuclear shuttle protein gene (DNA-B)
Pop-1	Entire population of ToLCPalV
Pop-2	ToLCPalV isolates from the Indo–Pak subcontinent
Pop-3	ToLCPalV isolates from the Middle East
MSA	Multiple sequence alignments
MEGA11	Molecular Evolutionary Genetics Analysis version 11
ClustalW	A widely used multiple sequence alignment program
SDT	Species demarcation tool
TempEst	Temporal Exploration of Sequences and Trees
BEAST	Bayesian Evolutionary Analysis Sampling Trees
HPD	Highest probability density
ML	Maximum likelihood
TN93	Tamura-Nei 1993 (nucleotide substitution model)
G+I	Gamma distribution plus invariant sites
iTOL	Interactive Tree Of Life
BEAUti2	Bayesian Evolutionary Analysis Utility—version 2
MCMC	Markov Chain Monte Carlo
ESS	Effective sample sizes
MCC	Maximum clade credibility
MJ	Median-joining
popART	Population Analysis with Reticulate Trees
DnaSP	DNA Sequence Polymorphism
π	Nucleotide diversity
H	Number of haplotypes
Hd	Haplotype diversity
S	Number of polymorphic/segregating sites
θw	Watterson’s theta
k	Average number of nucleotide differences between sequences
Eta	Total number of mutations
TD	Tajima’s D
FLD	Fu and Li’s D
NSSY	Nucleotide substitution rate per site per year
CoP1/2/3	Codon position 1/2/3
dN	Non-synonymous substitutions
dS	Synonymous substitutions
FUBAR	Fast, Unconstrained Bayesian AppRoximation
SLAC	Single-Likelihood Ancestor Counting
PS	Positively selected sites
NS	Negatively selected sites
RDP	Recombination Detection Program
GARD	Genetic Algorithm Recombination Detection
