# Supplementary material for: Genetic Diversity, Population Structure, and Cross-Border Dispersal Patterns of Tomato Leaf Curl Palampur Virus in South and West Asia
Source: Viruses. 2025 May 6;17(5):678. doi: 10.3390/v17050678 (PMC12115635; doi:10.3390/v17050678)
Supplement: Supplementary file 1 [file viruses-17-00678-s001.zip › viruses-3606266-supplementary.pdf]

(A)

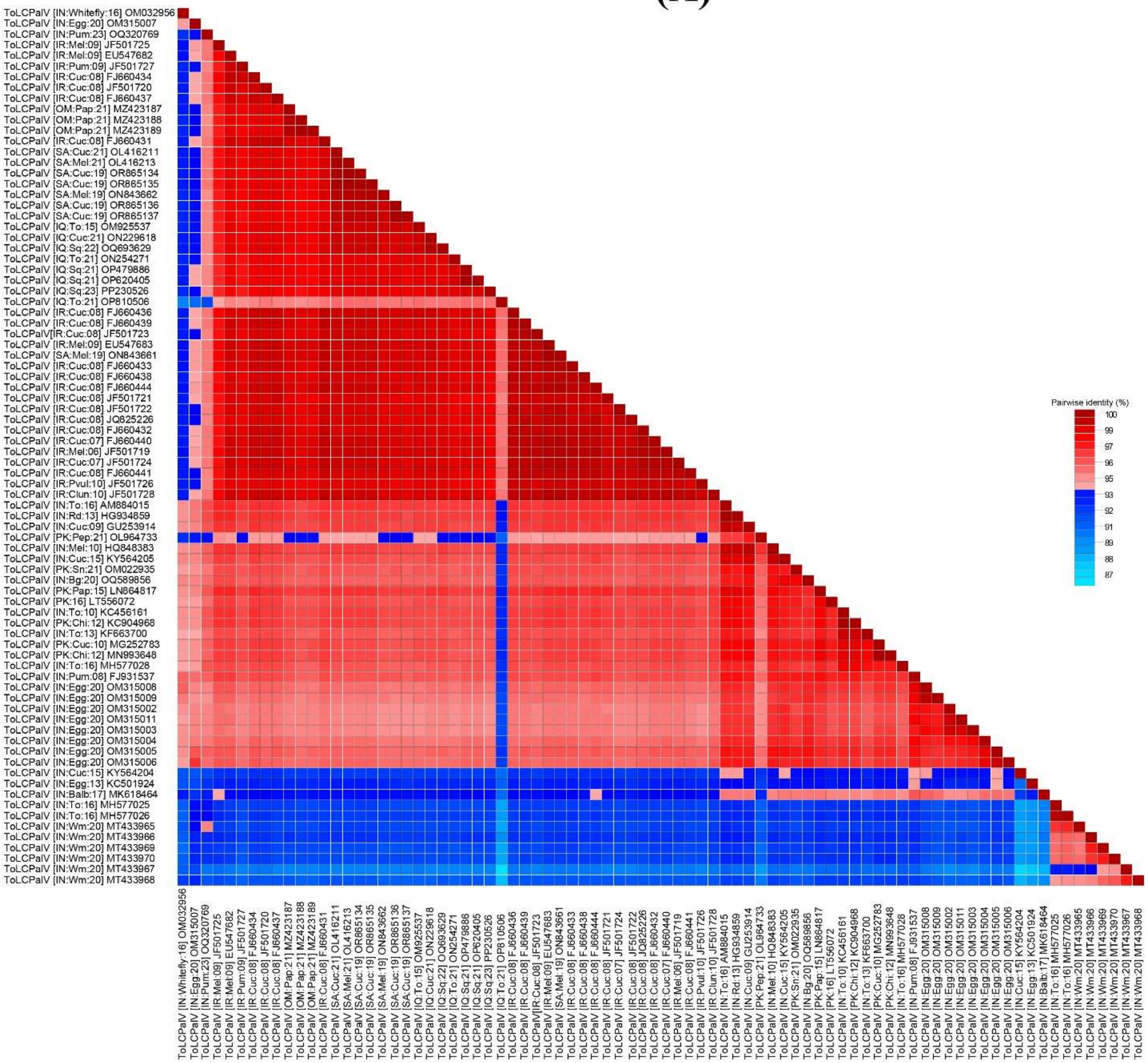
ToLCPalV [IN:Whitefly:16] OM032956
ToLCPalV [IN:Egg:20] OM315007
ToLCPalV [IN:Pum:23] OQ320769
ToLCPalV [IR:Mel:09] JF501725
ToLCPalV [IR:Mel:09] EU547682
ToLCPalV [IR:Pum:09] JF501727
ToLCPalV [IR:Cuc:08] FJ660434
ToLCPalV [IR:Cuc:08] JF501720
ToLCPalV [IR:Cuc:08] FJ660437
ToLCPalV [OM:Pap:21] MZ423187
ToLCPalV [OM:Pap:21] MZ423188
ToLCPalV [OM:Pap:21] MZ423189
ToLCPalV [IR:Cuc:08] FJ660431
ToLCPalV [SA:Cuc:21] OL416211
ToLCPalV [SA:Mel:21] OL416213
ToLCPalV [SA:Cuc:19] OR865134
ToLCPalV [SA:Cuc:19] OR865135
ToLCPalV [SA:Mel:19] ON843662
ToLCPalV [SA:Cuc:19] OR865136
ToLCPalV [SA:Cuc:19] OR865137
ToLCPalV [IQ:To:15] OM925537
ToLCPalV [IQ:Cuc:21] ON229618
ToLCPalV [IQ:Sq:22] OQ693629
ToLCPalV [IQ:To:21] ON254271
ToLCPalV [IQ:Sq:21] OP479886
ToLCPalV [IQ:Sq:21] OP620405
ToLCPalV [IQ:Sq:23] PP230526
ToLCPalV [IQ:To:21] OP810506
ToLCPalV [IR:Cuc:08] FJ660436
ToLCPalV [IR:Cuc:08] FJ660439
ToLCPalV[IR:Cuc:08] JF501723
ToLCPalV [IR:Mel:09] EU547683
ToLCPalV [SA:Mel:19] ON843661
ToLCPalV [IR:Cuc:08] FJ660433
ToLCPalV [IR:Cuc:08] FJ660438
ToLCPalV [IR:Cuc:08] FJ660444
ToLCPalV [IR:Cuc:08] JF501721
ToLCPalV [IR:Cuc:08] JF501722
ToLCPalV [IR:Cuc:08] JQ825226
ToLCPalV [IR:Cuc:08] FJ660432
ToLCPalV [IR:Cuc:07] FJ660440
ToLCPalV [IR:Mel:06] JF501719
ToLCPalV [IR:Cuc:07] JF501724
ToLCPalV [IR:Cuc:08] FJ660441
ToLCPalV [IR:Pvul:10] JF501726
ToLCPalV [IR:Clun:10] JF501728
ToLCPalV [IN:To:16] AM884015
ToLCPalV [IN:Rd:13] HG934859
ToLCPalV [IN:Cuc:09] GU253914
ToLCPalV [PK:Pep:21] OL964733
ToLCPalV [IN:Mel:10] HQ848383
ToLCPalV [IN:Cuc:15] KY564205
ToLCPalV [PK:Sn:21] OM022935
ToLCPalV [IN:Bg:20] OQ589856
ToLCPalV [PK:Pap:15] LN864817
ToLCPalV [PK:16] LT556072
ToLCPalV [IN:To:10] KC456161
ToLCPalV [PK:Chi:12] KC904968
ToLCPalV [IN:To:13] KF663700
ToLCPalV [PK:Cuc:10] MG252783
ToLCPalV [PK:Chi:12] MN993648
ToLCPalV [IN:To:16] MH577028
ToLCPalV [IN:Pum:08] FJ931537
ToLCPalV [IN:Egg:20] OM315008
ToLCPalV [IN:Egg:20] OM315009
ToLCPalV [IN:Egg:20] OM315002
ToLCPalV [IN:Egg:20] OM315011
ToLCPalV [IN:Egg:20] OM315003
ToLCPalV [IN:Egg:20] OM315004
ToLCPalV [IN:Egg:20] OM315005
ToLCPalV [IN:Egg:20] OM315006
ToLCPalV [IN:Cuc:15] KY564204
ToLCPalV [IN:Egg:13] KC501924
ToLCPalV [IN:Balb:17] MK618464
ToLCPalV [IN:To:16] MH577025
ToLCPalV [IN:To:16] MH577026
ToLCPalV [IN:Wm:20] MT433965
ToLCPalV [IN:Wm:20] MT433966
ToLCPalV [IN:Wm:20] MT433969
ToLCPalV [IN:Wm:20] MT433970
ToLCPalV [IN:Wm:20] MT433967
ToLCPalV [IN:Wm:20] MT433968
Pairwise identity (%)
100
99
97
96
95
93
92
91
89
88
87

(B)

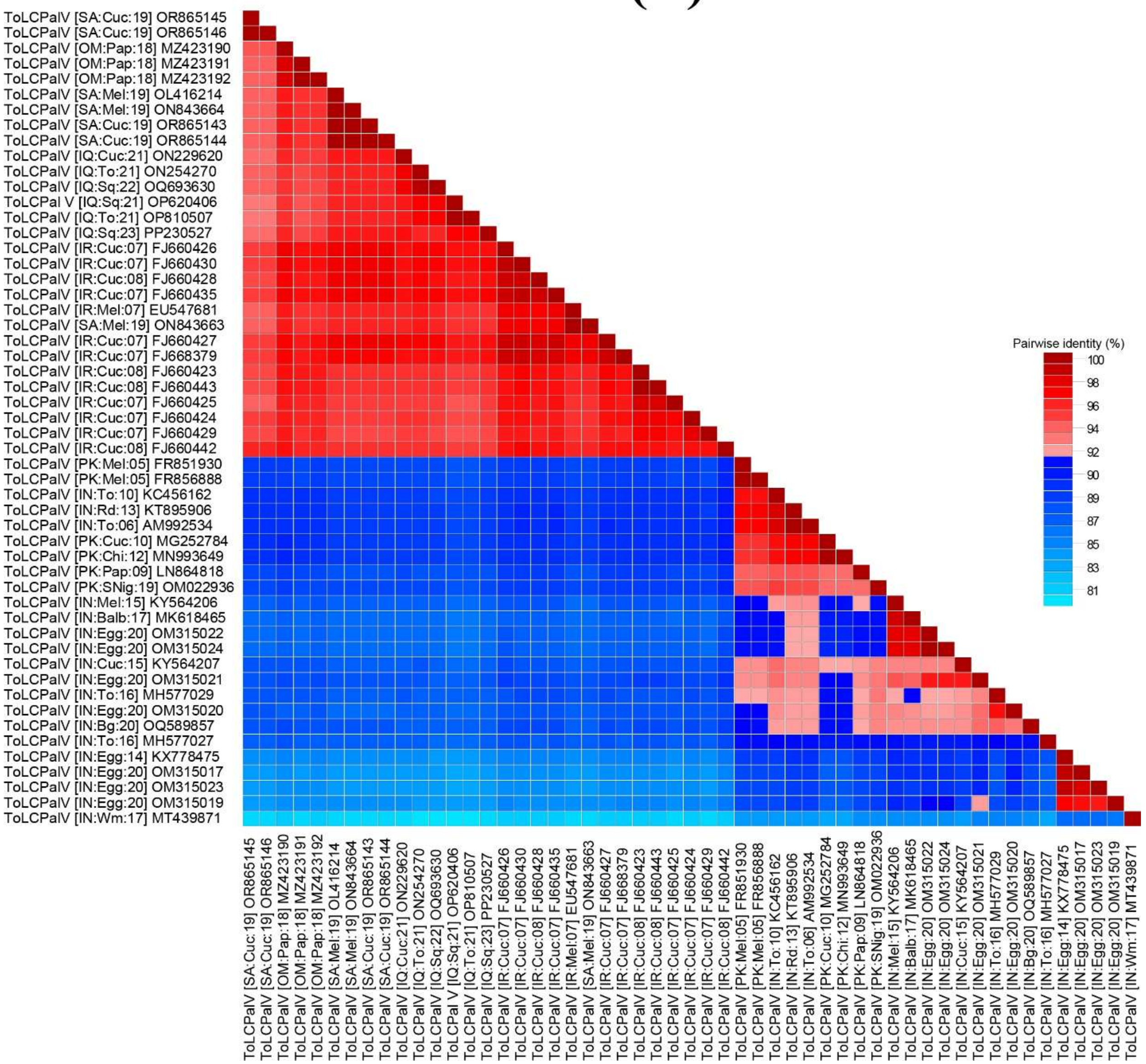
ToLCPalV [SA:Cuc:19] OR865145
ToLCPalV [SA:Cuc:19] OR865146
ToLCPalV [OM:Pap:18] MZ423190
ToLCPalV [OM:Pap:18] MZ423191
ToLCPalV [OM:Pap:18] MZ423192
ToLCPalV [SA:Mel:19] OL416214
ToLCPalV [SA:Mel:19] ON843664
ToLCPalV [SA:Cuc:19] OR865143
ToLCPalV [SA:Cuc:19] OR865144
ToLCPalV [IQ:Cuc:21] ON229620
ToLCPalV [IQ:To:21] ON254270
ToLCPalV [IQ:Sq:22] OQ693630
ToLCPal V [IQ:Sq:21] OP620406
ToLCPalV [IQ:To:21] OP810507
ToLCPalV [IQ:Sq:23] PP230527
ToLCPalV [IR:Cuc:07] FJ660426
ToLCPalV [IR:Cuc:07] FJ660430
ToLCPalV [IR:Cuc:08] FJ660428
ToLCPalV [IR:Cuc:07] FJ660435
ToLCPalV [IR:Mel:07] EU547681
ToLCPalV [SA:Mel:19] ON843663
ToLCPalV [IR:Cuc:07] FJ660427
ToLCPalV [IR:Cuc:07] FJ668379
ToLCPalV [IR:Cuc:08] FJ660423
ToLCPalV [IR:Cuc:08] FJ660443
ToLCPalV [IR:Cuc:07] FJ660425
ToLCPalV [IR:Cuc:07] FJ660424
ToLCPalV [IR:Cuc:07] FJ660429
ToLCPalV [IR:Cuc:08] FJ660442
ToLCPalV [PK:Mel:05] FR851930
ToLCPalV [PK:Mel:05] FR856888
ToLCPalV [IN:To:10] KC456162
ToLCPalV [IN:Rd:13] KT895906
ToLCPalV [IN:To:06] AM992534
ToLCPalV [PK:Cuc:10] MG252784
ToLCPalV [PK:Chi:12] MN993649
ToLCPalV [PK:Pap:09] LN864818
ToLCPalV [PK:SNig:19] OM022936
ToLCPalV [IN:Mel:15] KY564206
ToLCPalV [IN:Balb:17] MK618465
ToLCPalV [IN:Egg:20] OM315022
ToLCPalV [IN:Egg:20] OM315024
ToLCPalV [IN:Cuc:15] KY564207
ToLCPalV [IN:Egg:20] OM315021
ToLCPalV [IN:To:16] MH577029
ToLCPalV [IN:Egg:20] OM315020
ToLCPalV [IN:Bg:20] OQ589857
ToLCPalV [IN:To:16] MH577027
ToLCPalV [IN:Egg:14] KX778475
ToLCPalV [IN:Egg:20] OM315017
ToLCPalV [IN:Egg:20] OM315023
ToLCPalV [IN:Egg:20] OM315019
ToLCPalV [IN:Wm:17] MT439871
Pairwise identity (%)
100
98
96
94
92
90
89
87
85
83
81

Figure S1: Pairwise nucleotide sequence identities were calculated using the Species Demarcation Tool (SDT v. 1.2) to construct species demarcation matrices for **(A)** full-length tomato leaf curl Palampur virus (ToLCPalV) DNA-A and **(B)** ToLCPalV DNA-B. Results were visualized in a color-coded matrix, where red, green, and blue shading represented species and strain demarcation thresholds for begomovirus components, consistent with the criteria established by Muhire et al. (2013).

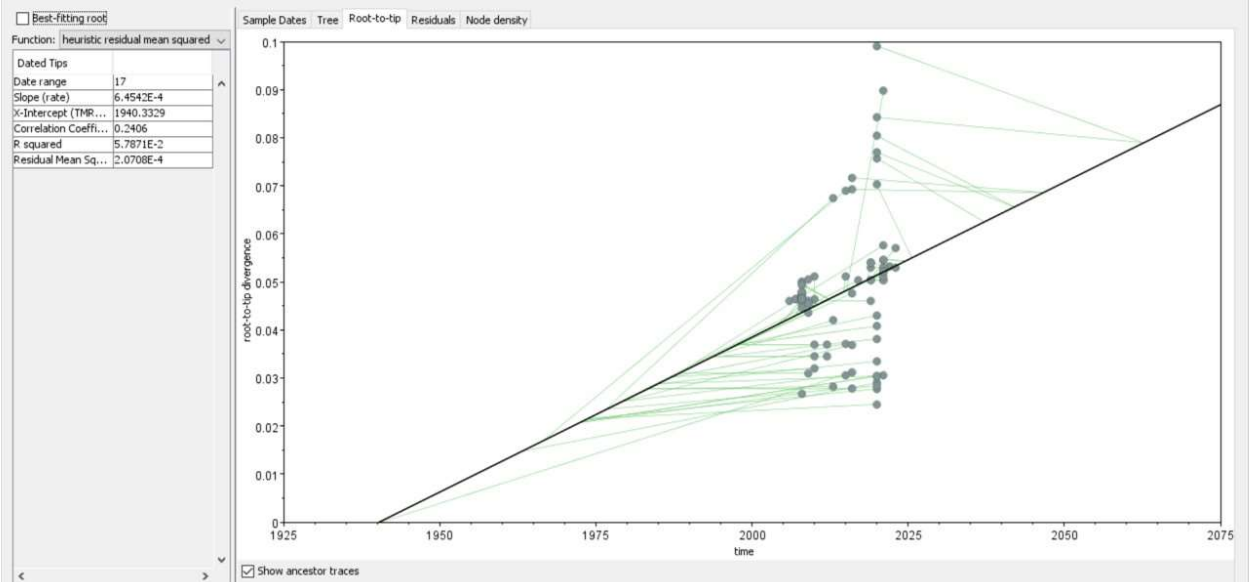

Figure S2: Root-to-tip regression analysis of the full-length ToLCPalV DNA-A, depicting the relationship between the root-to-tip genetic distance (values on Y-axis) and the sampling date (X-axis). Each circular point represents a unique isolate. The black line represents the best-fit linear regression, with statistics including the slope (rate of evolution), TMRCA (Time to Most Recent Common Ancestor, x-intercept), correlation coefficient (R), R-squared value, and residual mean square error displayed in the top left box.

Table S1: Complete dataset of ToLCPalV DNA-A and DNA-B isolates, including their associated plant host species, year of sample collection, and location of identification, along with corresponding geographical coordinates.

No.	Country	Host		Accession		Year	Location	Coordinates
		**Common name**	**Botanical name**	**DNA-A**	**DNA-B**			
1.	India	Watermelon	*Citrullus lanatus*	MT433965	MT439871	2020	Gogak, Karnatka	16°09'33.2"N 74°48'56.0"E
2.				MT433966	-	2020	Chikkaballapur, Karnataka	13°26'10.3"N 77°43'51.4"E
3.				MT433967	-	2020	Bengaluru, Karnataka	12°58'48.2"N 77°35'55.1"E
4.				MT433968	-	2020	Chamarajanagar, Karnataka	11°55'34.3"N 76°56'37.3"E
5.				MT433969	-	2020	Muddebihal, Vijayapura, Karnataka	16°20'22.5"N 76°07'44.7"E
6.				MT433970	-	2020	Raichur, Karnataka	16°12'57.7"N 77°21'23.6"E
7.		Squash	*Cucurbita pepo*	FJ931537	-	2008	Varanasi, Uttar Pradesh	25°19'04.9"N 82°58'26.2"E
8.		Bitter cucumber	*Cucumis callosus*	GU253914	-	2009	Lakhimpur, U. P	27°56'48.4"N 80°46'42.9"E
9.		Cucumber	*Cucumis sativus*	KY564205	KY564207	2015	Varanasi, Uttar Pradesh	25°19'04.9"N 82°58'26.2"E
10.		Melon	*Cucumis melo*	HQ848383	-	2010	Allipur, Uttar Pradesh	27°41'19.7"N 78°44'18.4"E
11.				KY564204	KY564206	2015	Varanasi, Uttar Pradesh	25°19'04.9"N 82°58'26.2"E
12.		Tomato	*Solanum lycopersicum*	KC456161	KC456162	2010	Punjab	30°41'21.4"N 75°14'36.9"E
13.				MH577025	MH577027	2016	Surendranagar, Gujarat	22°43'34.9"N 71°38'01.2"E
14.				MH577026	MH577029	2016	Surendranagar, Gujarat	22°43'34.9"N 71°38'01.2"E
15.				MH577028	-	2016	Bundi, Rajasthan	25°26'00.3"N 75°38'51.6"E
16.				KF663700	-	2013	Rauke, Punjab	30°39'33.9"N 75°16'03.9"E

17.				AM884015	AM992534	2013	Palampur, Himachal Pradesh	32°06'49.5"N 76°32'20.8"E
18.		Indian spinach	*Basella alba*	MK618464	MK618465	2017	Varanasi, Uttar Pradesh	25°19'04.9"N 82°58'26.2"E
19.		Whitefly	*Bemisia tabaci*	OM032956	-	2016	Varanasi, Uttar Pradesh	25°19'04.9"N 82°58'26.2"E
20.		Eggplant	*S. melongena*	OM315002	OM315017	2020	Varanasi, Uttar Pradesh	25°19'04.9"N 82°58'26.2"E
21.				OM315003	OM315018	2020	Mirzapur, Uttar Pradesh	25°08'10.1"N 82°33'49.8"E
22.				OM315004	OM315019	2020	Mirzapur, Uttar Pradesh	25°08'10.1"N 82°33'49.8"E
23.				OM315005	OM315020	2020	Rajasthan, Udaipur	24°35'22.3"N 73°42'32.4"E
24.				OM315006	OM315021	2020	Rajasthan, Udaipur	24°35'22.3"N 73°42'32.4"E
25.				OM315007	OM315022	2020	Bihar, Samastipur	25°51'25.2"N 85°47'20.6"E
26.				OM315008	OM315023	2020	Bihar, Samastipur	25°51'25.2"N 85°47'20.6"E
27.				OM315009	OM315024	2020	Ludihana, Punjab	30°54'29.6"N 75°50'46.5"E
28.				OM315011	-	2020	Varanasi, Uttar Pradesh	25°19'04.9"N 82°58'26.2"E
29.				KC501924	KX778475	2012	Mirzapur, Uttar Pradesh	25°08'10.1"N 82°33'49.8"E
30.		Buttercup squash	*Cucurbita maxima*	OQ320769	-	2022	Bhojpur, District Bhopal	23°05'59.3"N 77°34'47.5"E
31.		Bitter gourd	*Momordica charantia*	OQ589856	OQ589857	2020	Pusa, New Delhi	28°38'15.8"N 77°09'25.1"E
32.		Rumex	*Rumex crispus*	HG934859	KT895906	2013	Bandla, Palampur	32°07'55.7"N 76°32'21.6"E
33.	Pakistan	Chilli	*Capsicum frutescens*	KC904968	-	2012	Lahore, Punjab	31°28'45.3"N 74°15'60.0"E
34.		Pepper		MN993648	MN993649	2012	Lahore, Punjab	31°31'50.2"N 74°21'11.4"E

35.			*Capsicum annuum*	OL964733	-	2021	Khanewal, Punjab	30°17'13.6"N 71°55'59.3"E
36.		Tomato	*S. lycopersicum*	LT556072	-	2016	Swat, KPK	34°48'26.3"N 72°21'39.6"E
37.		Papaya	*Carica papaya*	LN864817	LN864818	2015	Faisalabad, Punjab	31°23'42.0"N 73°01'39.0"E
38.		Mako	*Solanum nigrum*	OM022935	OM022936	2021	Lahore, Punjab	31°28'45.3"N 74°15'60.0"E
39.		Cucumber	*C. sativus*	MG252783	MG252784	2010	Lahore, Punjab	31°28'45.3"N 74°15'60.0"E
40.		Melon	*C. melo*		FR851930	2005	Sahiwal, Punjab	30°40'11.9"N 73°06'35.3"E
41.					FR856888	2005	Vehari, Punjab	30°02'41.7"N 72°20'40.3"E
42.	Oman	Papaya	*C. papaya*	MZ423187	MZ423190	2018	Muscat, Oman	23°35'39.0"N 58°22'38.4"E
43.				MZ423188	MZ423191	2018		
44.				MZ423189	MZ423192	2018		
45.	Saudi Arabia	Melon	*C. melo*	OL416213		2019	Al Hofuf, Al-Ahsa	25°22'58.7"N 49°35'25.1"E
46.				ON843661	ON843663	2019	Al-Qatif	26°34'43.3"N 49°59'59.5"E
47.				ON843662	ON843664	2019	Al-Qatif	26°34'43.3"N 49°59'59.5"E
48.		Cucumber	*C. sativus*	OR865134	OR865143	2019	Al-Qatif	26°34'43.3"N 49°59'59.5"E
49.				OL416211	OL416214	2019	Al Hofuf, Al-Ahsa	25°22'58.7"N 49°35'25.1"E
50.				OR865135	OR865144	2019	Al-Qatif	26°34'43.3"N 49°59'59.5"E
51.				OR865136	OR865145	2019	Al Hofuf, Al-Ahsa	25°22'58.7"N 49°35'25.1"E
52.				OR865137	OR865146	2019	Al Hofuf, Al-Ahsa	25°22'58.7"N 49°35'25.1"E
53.	Iraq	Cucumber	*C. sativus*	ON229618	ON229620	2021	Babylon	32°28'34.5"N 44°25'22.8"E
54.		Tomato	*S. lycopersicum*	OM925537	-	2015	Dhi-qar	31°12'41.1"N 46°20'19.9"E
55.				ON254271	ON254270	2021	Karbala	32°36'16.3"N 44°01'03.7"E
56.				OP810506	OP810507	2021	Kufa	32°02'56.2"N 44°22'13.5"E
57.		Squash	*C. pepo*	OP479886	-	2021	Yusufiya	33°04'46.8"N 44°15'08.7"E
58.				PP230526	PP230527	2023	Baghdad	33°18'58.2"N 44°21'06.4"E
59.				OP620405	OP620406	2021	Baghdad	33°18'58.2"N 44°21'06.4"E
60.				OQ693629	OQ693630	2022	Baghdad	33°18'58.2"N 44°21'06.4"E

61.	Iran	Tomato	*S. lycopersicum*	EU547682	EU547681	2006	Rudan	27°39'49.3"N 57°05'44.6"E
62.		Melon	*C. melo*	EU547683	-	2007	Jiroft, Kerman	28°40'47.1"N 57°44'42.3"E
63.		Cucumber	*C. sativus*	FJ660431	FJ660423	2008	Jiroft, Kerman	28°40'47.1"N 57°44'42.3"E
64.				FJ660432	FJ660424	2007	Jiroft, Kerman	28°40'47.1"N 57°44'42.3"E
65.				FJ660433	FJ660425	2007	Kerman	30°17'08.8"N 57°04'54.1"E
66.				FJ660434	FJ660426	2007	Kahnooj, Kerman	27°56'30.3"N 57°41'58.5"E
67.				FJ660436	FJ660427	2007	Jiroft, Kerman	28°40'47.1"N 57°44'42.3"E
68.				FJ660437	FJ660428	2008		
69.				FJ660438	FJ660429	2008		
70.				FJ660439	FJ660430	2008		
71.				FJ660440	FJ660435	2008		
72.				FJ660441	FJ660442	2008		
73.				FJ660444	FJ660443	2008		
74.					FJ668379	2008		
75.		Melon	*C. melo*	JF501719	-	2006	Jiroft, Kerman	28°40'47.1"N 57°44'42.3"E
76.				JF501725	-	2009	Iranshahr, Sistan	27°12'13.6"N 60°41'03.5"E
77.		Cucumber	*C. sativus*	JF501720	-	2008	Jiroft, Kerman	28°40'47.1"N 57°44'42.3"E
78.				JF501721	-	2008		
79.				JF501722	-	2008		
80.				JF501723	-	2008		
81.				JF501724	-	2007		
82.				JQ825226	-	2008	Hormozgan	27°41'32.4"N 56°17'31.6"E
83.		Common bean	*Phaseolus vulgaris*	JF501726	-	2010	Hormozgan	27°41'32.4"N 56°17'31.6"E
84.		Squash	*C. pepo*	JF501727	-	2009	Jiroft, Kerman	28°40'47.1"N 57°44'42.3"E
85.		Watermelon	*Citrulus lunatus*	JF501728	-	2010	Khash, Sistan	28°13'14.1"N 61°12'42.6"E

Table S2: Estimation of genetic diversity and neutrality test indices for ToLCPalV DNA-A and DNA-B and their encoded ORFs in Pop-1 dataset

**Virus components**	**Number seq**	**InDel sites**	**S**	**Eta (h)**	**Number of variants***	**Hd**	**π**	**k**	**h**	**θw**	**Neutrality test**	
											**TD**	**FLD**
ToLCPV DNA-A	82	49	1012	1299	380/154/37	0.99	0.04	113.07	74	260.96	-1.97	-3.78
ToLCPV_DNAB	53	71	1179	1601	556/258/67	0.99	0.09	25.50	44	259.80	-1.05	-1.04
ToLCPV DNA-A-AC1	82	11	464	608	156/67/22	0.99	0.04	42.02	68	122.14	-2.27	-4.23
ToLCPV DNA-A-AC2	82	3	147	174	44/13/1	0.99	0.02	10.07	57	34.96	-2.42	-5.08
ToLCPV DNA-A-AC3	82	7	149	181	49/19/1	0.96	0.02	9.61	53	36.36	-2.51	-5.26
ToLCPV DNA-A-AC4	82	2	79	104	18/9/2	0.80	0.02	4.07	29	20.89	-2.71	-5.05
ToLCPV DNA-A-AV1	82	13	276	361	125/52/11	0.99	0.05	43.91	65	72.52	-1.3	-2.15
ToLCPV DNA-A-AV2	82	3	98	119	60/15/2	0.96	0.04	14.63	47	23.91	-1.3	-0.41
ToLCPV DNA-B-BV1	53	3	324	419	153/57/13	0.98	0.08	62.93	35	92.33	-1.15	-1.45
ToLCPV DNA-B-BC1	53	18	285	378	166/50/20	0.98	0.07	60.38	34	83.30	-0.99	0.08

* Parsimony informative sites (two variants/three variants/four variants)

Table S3: Estimation of genetic diversity and neutrality test indices for ToLCPalV DNA-A and DNA-B and their encoded ORFs in Pop-2 dataset

Virus components	Number seq	InDel sites	S	Eta (h)	Number of variants*	Hd	π	k	h	θw	Neutrality test	
											TD	FLD
ToLCPV DNA-A	39	47	891	1064	355/98/20	1.00	0.05	148.07	37	251.66	-1.55	-2.43
ToLCPV_DNAB	24	65	901	1115	412/142/23	0.99	0.09	230.99	22	298.58	-0.92	-0.96
ToLCPV DNA-A-AC1	39	15	389	468	131/37/11	0.99	0.05	53.23	34.00	110.69	-1.95	-3.03
ToLCPV DNA-A-AC2	39	5	125	141	39/8/0	0.97	0.04	14.96	29.00	33.35	-2.04	-3.36
ToLCPV DNA-A-AC3	39	6	134	154	44/13/1	0.98	0.04	16.64	30.00	36.42	-2.02	-3.16
ToLCPV DNA-A-AC4	39	4	54	63	9/2/0	0.87	0.03	4.24	18.00	14.90	-2.59	-4.36
ToLCPV DNA-A-AV1	39	5	671	981	116/120/25	0.99	0.13	96.52	34.00	232.03	-2.20	-4.00
ToLCPV DNA-A-AV2	39	3	90	104	63/11/1	0.98	0.07	23.79	28.00	24.60	-0.12	0.50
ToLCPV DNA-B-BV1	24	3	226	325	103/41/4	0.96	0.07	57.06	15	71.2	-1.3	-1.5
ToLCPV DNA-B-BC1	24	18	241	297`	146/39/7	0.982	0.086	73.11	33	64.5	-0.59	0.52

Table S4: Estimation of genetic diversity and neutrality test indices for ToLCPalV DNA-A and DNA-B and their encoded ORFs in Pop-3 dataset

Virus components	Number seq	InDel sites	S	Eta (h)	Number of variants*	Hd	π	k	h	θw	Neutrality test	
											TD	FLD
ToLCPV DNA-A	43	2	361	399	98/14/2	0.99	0.01	34.41	38	92.22	-2.32	-4.20
ToLCPV_DNAB	29	8	522	603	297/64/5	0.99	0.04	97.28	22	153.55	-1.44	-0.53
ToLCPV DNA-A-AC1	43	0	206	234	44/12/2	0.99	0.02	17.36	35	54.08	-2.50	-4.64
ToLCPV DNA-A-AC2	43	0	32	34	10/1/0	0.96	0.01	3.34	27	7.86	-1.98	-3.27
ToLCPV DNA-A-AC3	43	0	25	25	8/0/0	0.87	0.01	2.36	22	5.78	-1.98	-3.27
ToLCPV DNA-A-AC4	43	7	42	43	4/1/0	0.55	0.01	2.38	12	9.94	-2.66	-5.31
ToLCPV DNA-A-AV1	43	2	75	79	27/1/0	0.98	0.01	7.18	31	18.26	-2.18	-3.24
ToLCPV DNA-A-AV2	43	0	22	22	8/0/0	0.90	0.01	1.94	19	5.08	-2.04	-2.88
ToLCPV DNA-B-BV1	29	0	93	100	61/5/0	0.97	0.02	17.13	20	25.46	-1.26	-0.19
ToLCPV DNA-B-BC1	29	0	110	125	60/11/1	0.97	0.02	20.19	20	31.83	-1.41	-0.80

Table S5: Mean substitution and codon position mutation rate of the ToLCPalV Pop-1 and their encoded ORFs

**Virus components**	Clock type	Mean nt substitution rate (site^{-1} year^{-1})	At 95% HPD interval	CoP1 mu	CoP2 mu	CoP3 mu
DNA-A Pop-1	Strict	7.63×10^{-4} (7)	6.513×10^{-4}, 8.858×10^{-4}			
	Relaxed	8.04×10^{-4} (3)	6.270×10^{-4}, 9.961×10^{-4}			
DNA-B Pop-1	Strict	6.74×10^{-4} (7)	5.341×10^{-4}, 8.545×10^{-4}			
	Relaxed	7.08×10^{-4} (5)	3.371×10^{-4}, 5.605×10^{-4}			
ToLCPV DNA-A-AC1	Strict	7.34×10^{-4} (8)	5.933×10^{-4}, 8.980×10^{-4}	0.901 (383)	0.946 (747)	1.153 (484)
	Relaxed	8.42×10^{-4} (4)	5.9378×10^{-4}, 1.232×10^{-3}	0.922 (407)	0.932 (358)	1.147 (46)
ToLCPV DNA-A-AC2	Strict	3.25×10^{-4} (23)	2.411×10^{-4}, 4.164×10^{-4}	1.127 (759)	0.667 (901)	1.207 (264)
	Relaxed	3.50×10^{-4} (9)	2.354×10^{-4}, 4.804×10^{-4}	1.175 (474)	0.649 (757)	1.178 (264)
ToLCPV DNA-A-AC3	Strict	4.32×10^{-4} (31)	3.193×10^{-4}, 5.609×10^{-4}	0.813 (676)	0718 (548)	1.469 (788)
	Relaxed	4.73×10^{-4} (9)	3.041×10^{-4}, 6.463×10^{-4}	0.84 (148)	0.691 (299)	1.469 (176)
ToLCPV DNA-A-AC4	Strict	3.05×10^{-4} (34)	2.011×10^{-4}, 4.178×10^{-4}	0.526 (441)	1.58 (608)	0.894 (712)
	Relaxed	3.35×10^{-4} (12)	1.991×10^{-4}, 5.032×10^{-4}	0.506 (636)	1.477 (546)	1.017 (812)
ToLCPV DNA-A-AV1	Strict	5.36×10^{-4} (13)	4.157×10^{-4}, 6.615×10^{-4}	1.24 (171)	0.383 (309)	1.377 (165)
	Relaxed	5.73×10^{-4} (7)	4.212×10^{-4}, 7.721×10^{-4}	1.245 (688)	0.385 (820)	137 (742)

ToLCPV DNA-A-AV2	Strict	2.59×10^{-4} (57)	1.818×10^{-4}, 3.494×10^{-4}	0.92 (848)	0.496 (705)	1.584 (670)
	Relaxed	2.71×10^{-4} (11)	1.719×10^{-4}, 3.995×10^{-4}	0.973 (901)	0.508 (798)	1.519 (548)
ToLCPV DNA-B-BV1	Strict	4.53×10^{-4} (24)	3.265×10^{-4}, 6.165×10^{-4}	1.762 (815)	0.702 (845)	0.533 (735)
	Relaxed	4.62×10^{-4} (8)	2.6×10^{-2}, 8.48×10^{-2}	1.733 (640)	0.707 (647)	0.517 (437)
ToLCPV DNA-B-BC1	Strict	3.48×10^{-4} (10)	2.135×10^{-4}, 9.398×10^{-4}	1.389 (538)	0.338 (414)	1.274 (550)
	Relaxed	4.73×10^{-4} (11)	1.350×10^{-4}, 2.837×10^{-4}	1.376 (499)	0.34 (548)	1.285 (643)

Table S6: Mean substitution and codon position mutation rate of the ToLCPalV Pop-2 and their encoded ORFs

**Virus components**	Clock type	Mean nt substitution rate (site^{-1} year^{-1}	At 95% HPD interval	CoP1 mu	CoP2 mu	CoP3 mu
DNA-A Pop-2	Strict	8.23×10^{-4} (12)	6.513×10^{-4}, 8.858×10^{-4}			
	Relaxed	7.49×10^{-4} (5)	4.488×10^{-4}, 9.657×10^{-4}			
DNA-B Pop-2	Strict	4.49×10^{-4} (11)	[5.341×10^{-4}, 8.545×10^{-4}			
	Relaxed	3.67×10^{-4} (12)	2.628×10^{-4}, 5.255×10^{-4}			
ToLCPV DNA-A-AC1	Strict	1.04×10^{-3} (20)	7.304×10^{-4}, 1.336×10^{-3}	0.962 (692)	0.838 (817)	1.201 (806)
	Relaxed	3.77×10^{-4} (13)	1.945×10^{-4}, 5.818×10^{-4}	0.932 (18)	0.83 (716)	1.239 (19)
ToLCPV DNA-A-AC2	Strict	2.78×10^{-4} (130)	1.842×10^{-4}, 3.797×10^{-4}	0.959 (669)	0941 (751)	1.1 (828)
	Relaxed	2.22×10^{-4} (17)	1.261×10^{-4}, 3.292×10^{-4}	0.96 (500)	0.947 (644)	1.093 (652)
ToLCPV DNA-A-AC3	Strict	3.74×10^{-4} (110)	2.505×10^{-4}, 5.059×10^{-4}	1.169 (475)	0884 (332)	0946 (435)
	Relaxed	2.90×10^{-4} (29)	1.698×10^{-4}, 4.179×10^{-4}	1.158 (837)	0.897 (611)	0.944 (836)
ToLCPV DNA-A-AC4	Strict	2.10×10^{-4} (164)	1.173×10^{-4}, 3.094×10^{-4}	0.963 (649)	0914 (769)	1.125 (762)
	Relaxed	1.60×10^{-4} (24)	7.886×10^{-5}, 2.417×10^{-4}	0.906 (367)	0.897 (692)	1.2 (740)
ToLCPV DNA-A-AV1	Strict	6.81×10^{-4} (27)	5.021×10^{-4}, 8.423×10^{-4}	0.485 (580)	0.465 (444)	2.05 (460)
	Relaxed	5.52×10^{-4} (11)	3.696×10^{-4}, 7.3862×10^{-4}	0.431 (525)	0.375 (369)	2.195 (339)
ToLCPV DNA-A-AV2	Strict	2.40×10^{-4} (130)	1.511×10^{-4}, 3.389×10^{-4}	0.918 (729)	0.569 (901)	1.512 (430)

	Relaxed	1.84×10^{-4} (26)	1.012×10^{-4}, 2.745×10^{-4}	0.994 (76)	0.586 (272)	1.421 (97)
ToLCPV DNA-B-BV1	Strict	3.80×10^{-4} (21)	2.280×10^{-4}, 5.646×10^{-4}	1.816 (713)	0.695 (901)	0.486 (901)
	Relaxed	2.84×10^{-4} (25)	1.746×10^{-4}, 4.037×10^{-4}	1.814 (625)	0.695 (413)	0.487 (669)
ToLCPV DNA-B-BC1	Strict	2.03×10^{-4} (98)	1.350×10^{-4}, 2.837×10^{-4}	1.342 (879)	0.352 (692)	1.308 (809)
	Relaxed	1.66×10^{-4} (32)	9.542×10^{-5}, 2.478×10^{-4}	1.358 (373)	0.36 (642)	1.283 (388)

Table S7: Mean substitution and codon position mutation rate of the ToLCPalV Pop-3 and their encoded ORFs

**Virus components**	Clock type	Mean nt substitution rate (site^{-1} year^{-1}	At 95% HPD interval	CoP1 mu	CoP2 mu	CoP3 mu
DNA-A Pop-3	Strict	7.06×10^{-4} (77)	5.864×10^{-4}, 8.310×10^{-4}			
	Relaxed	7.30×10^{-4} (74)	6.112×10^{-4}, 8.680×10^{-4}			
DNA-B Pop-3	Strict	8.81×10^{-4} (52)	7.250×10^{-4}, 1.048×10^{-3}			
	Relaxed	9.40×10^{-4} (32)	7.801×10^{-4}, 1.112×10^{-3}			
ToLCPV DNA-A-AC1	Strict	5.91×10^{-4} (210)	4.594×10^{-4}, 7.294×10^{-4}	0.586 (901)	0.416 (901)	2.001 (672)
	Relaxed	6.12×10^{-4} (126)	4.708×10^{-4}, 7.749×10^{-4}	0.624 (901)	0.433 (667)	1.945 (648)
ToLCPV DNA-A-AC2	Strict	2.97×10^{-4} (416)	1.754×10^{-4}, 4.175×10^{-4}	1.047 (759)	0.686 (631)	1.269 (498)
	Relaxed	3.12×10^{-4} (423)	1.968×10^{-4}, 4.528×10^{-4}	1.034 (318)	0.672 (565)	0.43 (385)
ToLCPV DNA-A-AC3	Strict	2.73×10^{-4} (442)	1.598×10^{-4}, 3.957×10^{-4}	0.784 (812)	0.591 (717)	1.629 (778)
	Relaxed	2.91×10^{-4} (309)	1.709×10^{-4}, 4.232×10^{-4}	0.769 (705)	0.594 (901)	1.641 (710)
ToLCPV DNA-A-AC4	Strict	4.75×10^{-4} (340)	2.828×10^{-4}, 6.969×10^{-4}	0.59 (776)	1.447 (836)	0.963 (852)
	Relaxed	5.19×10^{-4} (161)	3.006×10^{-4}, 7.647×10^{-4}	0.617 (627)	1.453 (654)	0.928 (379)
ToLCPV DNA-A-AV1	Strict	4.02×10^{-4} (246)	2.658×10^{-4}, 5.354×10^{-4}	1.987 (657)	0.44 (826)	0.57 (740)
	Relaxed	4.22×10^{-4} (221)	2.826×10^{-4}, 5.589×10^{-4}	1.998 (774)	0.439 (739)	0.559 (676)
ToLCPV DNA-A-AV2	Strict	2.10×10^{-4} (564)	1.046×10^{-4}, 2.979×10^{-4}	1.016 (481)	0.371 (486)	1.612 (292)

	Relaxed	1.84×10^{-4} (26)	1.130×10^{-4}, 3.213×10^{-4}	0.91 (320)	0.41 (166)	1.679 (376)
ToLCPV DNA-B-BV1	Strict	5.31×10^{-4} (91)	3.717×10^{-4}, 7.173×10^{-4}	0.55 (901)	0.542 (643)	1.912 (530)
	Relaxed	5.55×10^{-4} (125)	3.825×10^{-4}, 7.207×10^{-4}	0.558 (755)	0.547 (283)	1.899 (353)
ToLCPV DNA-B-BC1	Strict	5.61×10^{-4} (226)	4.114×10^{-4}, 7.217×10^{-4}	0.411 (705)	0.324 (838)	2.27 (708)
	Relaxed	5.92×10^{-4} (156)	3.636×10^{-4}, 8.245×10^{-4}	0.418 (808)	0.326 (705)	0.326 (705)

Table S8: Estimation of selection pressure on the ORFs encoded by ToLCPalV isolates in Pop-1

Virus components	Best model	Mean distance (d)	dN	dS	dN/dS	FUBAR Posterior probability (p≤0.9)		SLAC (p≤0.05)	
						PS	NS	PS	NS
ToLCPV DNA-A-AC1	HKY+G	0.042±0.003	0.0313	0.0836	0.374401914	2	102	0	51
ToLCPV DNA-A-AC2	K2+G	0.026±0.003	0.0191	0.0575	0.332173913	1	8	0	5
ToLCPV DNA-A-AC3	T92+G	0.028±0.003	0.0224	0.0679	0.329896907	2	17	0	8
ToLCPV DNA-A-AC4	K2+I	0.034±0.012	0.0444	0.0802	0.55361596	3	2	0	0
ToLCPV DNA-A-AV1	K2+G	0.064±0.006	0.0404	0.1748	0.231121281	2	108	0	62
ToLCPV DNA-A-AV2	K2+G	0.047±0.006	0.0362	0.1472	0.245923913	5	17	0	15
ToLCPV DNA-B-BV1	T92+G+I	0.086±0.006	0.0928	0.0714	1.299719888	3	121	1	45
ToLCPV DNA-B-BC1	HKY+G	0.078±0.009	0.0632	0.1469	0.430224643	1	123	1	58

Table S9: Estimation of selection pressure on the ORFs encoded by ToLCPalV isolates in Pop-2

**Virus components**	**Best model**	**Mean distance (d)**	**dN**	**dS**	**dN/dS**	**FUBAR Posterior probability (p≤0.9)**		**SLAC (p≤0.05)**	
						**PS**	**NS**	**PS**	**NS**
ToLCPV DNA-A-AC1	T92+G	0.051±0.003	0.0399	0.0945	0.422222222	3	88	0	45
ToLCPV DNA-A-AC2	K2+G	0.037±0.005	0.031	0.0703	0.440967283	2	4	0	1
ToLCPV DNA-A-AC3	T92+G	0.043±0.005	0.0403	0.0627	0.642743222	1	14	0	6
ToLCPV DNA-A-AC4	JC	0.17±0.008	0.0536	0.0979	0.547497446	0	0	0	0
ToLCPV DNA-A-AV1	T92+G	0.328±0.040	0.0635	0.4108	0.154576436	3	105	0	51
ToLCPV DNA-A-AV2	K2+G	0.080±0.018	0.059	0.2089	0.282431786	5	12	0	7
ToLCPV DNA-B-BV1	T92+G	0.077±0.009	0.0857	0.0638	1.343260188	2	82	0	35
ToLCPV DNA-B-BC1	HKY+G	0.097±0.005	0.0767	0.2055	0.37323601	1	105	0	22

Table S10: Estimation of selection pressure on the ORFs encoded by ToLCPalV isolates in Pop-3

**Virus components**	**Best model**	**Mean distance (d)**	**dN**	**dS**	**dN/dS**	**FUBAR Posterior probability (p≤0.9)**		**SLAC (p≤0.05)**	
						**PS**	**NS**	**PS**	**NS**
ToLCPV DNA-A-AC1	T92+G	0.018±0.002	0.0083	0.0555	0.14954955	2	45	0	11
ToLCPV DNA-A-AC2	JC+G	0.009±0.002	0.0067	0.0232	0.288793103	0	6	0	1
ToLCPV DNA-A-AC3	JC	0.008±0.002	0.0083	0.0195	0.425641026	0	5	0	0
ToLCPV DNA-A-AC4	JC	0.024±0.014	0.0352	0.0724	0.486187845	2	1	0	0
ToLCPV DNA-A-AV1	K2+G	0.010±0.002	0.0108	0.0118	0.915254237	0	13	0	2
ToLCPV DNA-A-AV2	JC	0.006±0.002	0.0047	0.0203	0.231527094	0	5	0	1
ToLCPV DNA-B-BV1	T92+G	0.022±0.003	0.0124	0.0585	0.211965812	2	19	0	5
ToLCPV DNA-B-BC1	K2+G	0.024±0.004	0.0096	0.0845	0.113609467	3	36	0	4
